# Efficacy of neoadjuvant endocrine therapy with CDK4/6 inhibitors in locally advanced breast cancer

**DOI:** 10.1093/oncolo/oyag032

**Published:** 2026-02-06

**Authors:** Mengqi Zhang, Mingxiao Li, Shihan Zhou, Mingxia Jiang, Jiaxuan Liu, Xue Yang, Ling Qin, Nilupai Abudureheiyimu, Xiuqing Shi, Lixi Li, Fengjuan Li, Xiuwen Guan, Fei Ma, Binghe Xu, Qiao Li

**Affiliations:** Department of Medical Oncology, Cancer Hospital, Chinese Academy of Medical Sciences and Peking Union Medical College, Beijing, 100021, China; Department of Medical Oncology, Cancer Hospital, Chinese Academy of Medical Sciences and Peking Union Medical College, Beijing, 100021, China; Department of Medical Oncology, Cancer Hospital, Chinese Academy of Medical Sciences and Peking Union Medical College, Beijing, 100021, China; Department of Medical Oncology, Cancer Hospital, Chinese Academy of Medical Sciences and Peking Union Medical College, Beijing, 100021, China; Department of Medical Oncology, Cancer Hospital, Chinese Academy of Medical Sciences and Peking Union Medical College, Beijing, 100021, China; Department of Medical Oncology, Cancer Hospital, Chinese Academy of Medical Sciences and Peking Union Medical College, Beijing, 100021, China; Department of Medical Oncology, Cancer Hospital, Chinese Academy of Medical Sciences and Peking Union Medical College, Beijing, 100021, China; Department of Medical Oncology, Cancer Hospital, Chinese Academy of Medical Sciences and Peking Union Medical College, Beijing, 100021, China; Department of Medical Oncology, Cancer Hospital, Chinese Academy of Medical Sciences and Peking Union Medical College, Beijing, 100021, China; Department of Medical Oncology, Cancer Hospital, Chinese Academy of Medical Sciences and Peking Union Medical College, Beijing, 100021, China; Department of Medical Oncology, Cancer Hospital, Chinese Academy of Medical Sciences and Peking Union Medical College, Beijing, 100021, China; Department of Medical Oncology, Cancer Hospital, Chinese Academy of Medical Sciences and Peking Union Medical College, Beijing, 100021, China; Department of Medical Oncology, Cancer Hospital, Chinese Academy of Medical Sciences and Peking Union Medical College, Beijing, 100021, China; Department of Medical Oncology, Cancer Hospital, Chinese Academy of Medical Sciences and Peking Union Medical College, Beijing, 100021, China; Department of Medical Oncology, Cancer Hospital, Chinese Academy of Medical Sciences and Peking Union Medical College, Beijing, 100021, China

**Keywords:** breast cancer, neoadjuvant therapy, endocrine treatment, CDK4/6 inhibitor

## Abstract

**Background:**

Neoadjuvant treatment for hormone receptor (HR)-positive breast cancer remains limited, particularly for tumors that are insensitive to neoadjuvant chemotherapy. This study aims to compare the efficacy of neoadjuvant endocrine therapy combined with CDK4/6 inhibitors to that of traditional neoadjuvant chemotherapy.

**Patients and methods:**

A total of 49 patients receiving neoadjuvant endocrine therapy plus CDK4/6 inhibitors and 210 receiving neoadjuvant chemotherapy were enrolled. Magnetic resonance imaging was performed to assess tumor responses every 2-3 cycles of treatment. Propensity score matching (PSM) was performed to balance baseline characteristics.

**Results:**

Both before and after PSM, the objective response rate (ORR) in the endocrine therapy group was comparable to that of the traditional chemotherapy group. After matching, the ORR was 64.6% (95% CI, 49.5-77.8) in the endocrine group and 56.3% (95% CI, 41.2-70.5) in the chemotherapy group (*P *= .532). A higher proportion of patients in the endocrine group achieved a pathological complete or near-complete response (Miller-Payne grades 4-5, 13.5% vs 10.4%) and post-treatment Ki67 < 5%, indicating a potential long-term benefit. Patients with ≥30% regression in maximal tumor diameter after 2 cycles of chemotherapy were considered chemotherapy sensitive. Among patients with tumors less sensitive to chemotherapy, sequential treatment with neoadjuvant endocrine therapy plus CDK4/6 inhibitors significantly improved ORR (61.8% vs 32.4%, *P *= .028) and was associated with greater Ki67 reduction and improved Miller-Payne grades.

**Conclusion:**

Neoadjuvant endocrine therapy is a promising alternative for HR-positive breast cancer, especially for patients with poor responses to chemotherapy.

Implications for Practice HR+/HER2- breast cancer remains constrained by the limitations in current neoadjuvant treatment approaches and the therapeutic efficacy. For tumors that are non-responsive to conventional neoadjuvant chemotherapy, there is often a lack of effective alternative strategies. By conducting a retrospective analysis of the efficacy of neoadjuvant endocrine therapy, this study aimed to provide possible clinical insights and evidence for treatment selection.

## Introduction

Breast cancer has the highest incidence rate in females and is a leading cause of cancer-related mortality worldwide.^1^ Hormone receptor (HR)-positive breast cancer dominated with a proportion of approximately 80%, with HR-positive and human epidermal growth receptor 2 (HER2)-negative being the most prevalent molecular subtype, which is generally associated with a better overall prognosis, meanwhile carries a sustained risk of recurrence.^2^^,^^3^ By the advancement of endocrine therapy, HR-positive breast cancer has a significant improvement in outcomes over the past decades.^4^ On this basis, how to enable patients of this subtype to further benefit from treatment has become a new challenge.

Since the 1970s, neoadjuvant chemotherapy has begun to be applied before breast cancer surgery, aiming to downstage locally advanced (inoperable) population, and then subsequently used for operable (early) breast cancer to allow breast-conserving surgery.^5^ The approach to neoadjuvant therapy has also expanded from chemotherapy to targeted therapy, becoming more diverse.^6^ HER2-posivite breast cancer has definitely benefited from HER2-targeted monoclonal antibody neoadjuvant therapy, while triple-negative breast cancer (TNBC) is also seeing greater benefits from neoadjuvant therapy with the development of immune treatments in recent years.^6^^,^^7^ Meanwhile, the HR+/HER2- subtype is still primarily limited to chemotherapy during the neoadjuvant phase and had the lowest pathologic complete response (pCR) rate of 10%-20%, while endocrine therapy is more commonly applied in the postoperative adjuvant intensification phase.^8–10^ There is still significant potential for improving the effectiveness of neoadjuvant therapy in HR+/HER2- breast cancer.

The options for endocrine therapy typically include steroidal (exemestane) or non-steroidal (letrozole or anastrozole) aromatase inhibitors (AIs), selective estrogen receptor modulators (SERMs), such as tamoxifen or toremifene, and selective estrogen receptor degraders (SERDs), such as fulvestrant.^11^ Through various signaling pathways, endocrine therapy alters cellular metabolism in estrogen receptor (ER)-positive breast tumors by depriving the tumor of estrogen or blocking the activity of ERα, thereby preventing the proliferation of tumor cells.^2^^,^^12^ A standard course of endocrine therapy for 5-10 years in patients with early-stage breast cancer, or its combination with CDK4/6 inhibitors in patients with locally advanced or metastatic disease, can improve disease-free survival (DFS), progression-free survival (PFS), and overall survival (OS).^11^ In clinical practice, the use of endocrine therapy in the neoadjuvant setting is approached with caution and is generally limited to patients who are elderly, have significant comorbidities, psychiatric disorders, or other contraindications to surgery, which is restricted applicability. Recent clinical trials, such as WSG ADAPTcycle and VOG-01, have demonstrated that neoadjuvant endocrine therapy combined with CDK4/6 inhibitors achieves efficacy comparable to chemotherapy in HR+/HER2-breast cancer, with better safety.^13^^,^^14^ However, further evidence is needed to clarify their role in chemotherapy omission.

In contrast to the aforementioned that conduct the neoadjuvant endocrine therapy strategies directly, this study focused on evaluating neoadjuvant endocrine therapy in HR-positive patients, particularly those who show limited response to conventional neoadjuvant chemotherapy. By comparing and analyzing the efficacy of neoadjuvant chemotherapy and endocrine therapy, the study seeks to provide more effective and individualized therapeutic options for patients with HR-positive breast cancer.

## Patients and methods

### Patient selection

Breast cancer patients who received neoadjuvant endocrine therapy plus CDK4/6 inhibitors, with or without prior chemotherapy, at Cancer Hospital Chinese Academy of Medical Sciences, between May 2021 and March 2025 were enrolled in the endocrine therapy cohort. Patients received traditional neoadjuvant chemotherapy were collected in the chemotherapy cohort. Inclusion criteria include: (1) pathologically confirmed HR-positive, HER2-negative breast cancer (IHC 0-2+ with negative FISH); (2) female patients with early or locally advanced disease (stage I-III); and (3) complete clinical data and imaging-based efficacy evaluation. Exclusion criteria include: (1) incomplete pathological or efficacy data; (2) no neoadjuvant treatment; (3) ER ≤10% (biologically close to TNBC); and (4) distant metastasis or occult breast cancer with unmeasurable lesions.

### Study variables and response assessment

Baseline variables include age, menarche, menopause status, histology, grade, T stage, N stage, and Ki67/ER/HER2 expression. The primary endpoint was ORR per RECIST v1.1 (CR + PR). Second endpoints included the changes in Ki67 expression and Miller-Payne grading at surgery before and after treatments. And Miller-Payne grade 5 (all the infiltrating cancer cells in the original tumor bed have regressed) or 4 (the infiltrating cancer cells in the original tumor bed have regressed by more than 90%) were recognized as pCR and near-pCR.

Magnetic resonance imaging (MRI) was performed to assess tumor responses every 2-3 cycles of treatment. Tumors with ≥30% regression in maximal diameter after 2 cycles of chemotherapy were considered chemotherapy-sensitive.

### Propensity score matching

To reduce baseline imbalance, 1:1 nearest-neighbor propensity score matching (PSM) was performed using RStudio (v2023.12.1 + 402).

### Statistical analysis

Baseline comparisons used chi-square or Fisher’s exact test. 95% CI were calculated by the Clopper-Pearson method. ORR and significance testing were conducted using SPSS (v29.0.1.0). Forest plots and Sankey diagrams were generated using GraphPad Prism (v10) and RStudio respectively. A *P*-value <.05 was considered statistically significant.

## Results

### Patient characteristics at baseline

Patients with HR-positive breast cancer who received neoadjuvant endocrine therapy plus CDK4/6 inhibitors, with or without prior chemotherapy, at Cancer Hospital, Chinese Academy of Medical Sciences, between May 2021 and March 2025 were enrolled in the endocrine therapy cohort, while those who received traditional neoadjuvant chemotherapy were included in the chemotherapy cohort. A total of 49 patients were enrolled in the endocrine therapy group, and 210 patients were included in the chemotherapy group. The demographic and clinicopathological characteristics of patients in these groups are shown in Table 1. Before PSM, significant differences (*P *< .05) were observed between the 2 groups in terms of age at first diagnose, menopausal status, and grade. Specifically, elderly patients at first diagnose were more likely to receive neoadjuvant endocrine therapy (17.1% vs 38.8%, *P *= .001), while younger patients were more likely to receive traditional neoadjuvant chemotherapy (50.5% vs 26.5%, *P *= .001). Relatively, a higher proportion of postmenopausal patients received endocrine therapy compared to chemotherapy (59.2% vs 38.1%, *P *= .007). Additionally, patients with grade III were more likely to receive chemotherapy (19.0% vs 6.1%, *P *= .042). There were no statistically significant differences (*P *> .05) between the 2 types of neoadjuvant treatment in terms of age at menarche, histological type, T/N stage, Ki67 level, ER expression, or HER2 status at baseline. To reduce bias, PSM were performed at a 1:1 ratio, creating a matched cohort of 96 patients. After matching, there were no significant differences in the variables between the 2 groups (*P *> .05). The effect of PSM is shown in Figure S1 (See online supplementary material for a color version of this figure), and the matched list is shown in Table S1.

**Table 1.** Demographic and clinicopathological characteristics of patients in neoadjuvant endocrine therapy group and chemotherapy group before and after PSM.

	Before PSM			After PSM		
	Neoadjuvant endocrine therapy	Neoadjuvant chemotherapy	*P*	Neoadjuvant endocrine therapy	Neoadjuvant chemotherapy	*P*
	*N* = 49	*N* = 210		*N* = 48	*N* = 48	
**Age at first diagnose**			**.001**			**.815**
** ≤45**	13 (26.5%, 34 y-45 y)	106 (50.5%, 27 y-45 y)		13 (27.1%, 34 y-45 y)	15 (31.3%, 27 y-45 y)	
** 46-59**	17 (34.7%, 47 y-59 y)	68 (32.4%, 46 y-59 y)		16 (33.3%, 47 y-59 y)	13 (27.1%, 46 y-59 y)	
** ≥60**	19 (38.8%, 60 y-75 y)	36 (17.1%, 60 y-71 y)		19 (39.6%, 60 y-75 y)	20 (41.7%, 60 y-71 y)	
**Menarche age**			**.930**			**.649**
** ≤12**	6 (12.2%, 11 y-12 y)	30 (14.3%, 11 y-12 y)		6 (12.5%, 11 y-12 y)	3 (6.3%, 12 y)	
** 13-15**	33 (67.3%, 13 y-15 y)	137 (65.2%, 13 y-15 y)		32 (66.7%, 13 y-15 y)	34 (70.8%, 13 y-15 y)	
** ≥16**	137 (65.2%, 13 y-15 y)	43 (20.5%, 16 y-18 y)		10 (20.8%, 16 y-19 y)	11 (22.9%, 16 y-18 y)	
**Menopause**			**.007**			**1.000**
** Yes**	29 (59.2%)	80 (38.1%)		29 (60.4%)	28 (58.3%)	
** No**	20 (40.8%)	130 (61.9%)		19 (39.6%)	20 (41.7%)	
**Histology**			**.529**			**1.000**
** IDC**	42 (85.7%)	183 (87.1%)		42 (87.5%)	43 (89.6%)	
** Non-IDC**	7 (14.3%)	22 (10.5%)		6 (12.5%)	5 (10.4%)	
** NA**	0 (0%)	5 (2.4%)		-	-	
**Grade**			**.042**			**.794**
** I**	3 (6.1%)	5 (2.4%)		3 (6.3%)	2 (4.2%)	
** II**	42 (85.7%)	154 (73.3%)		42 (87.5%)	44 (91.7%)	
** III**	3 (6.1%)	40 (19.0%)		3 (6.3%)	2 (4.2%)	
** Others or NA**	1 (2.0%)	11 (5.2%)		-		
**T stage (tumor size, mm)**			**.532**			**.989**
** 1 (T ≤ 20)**	6 (12.2%)	21 (10.0%)		6 (12.5%)	6 (12.5%)	
** 2 (20 < T ≤ 50)**	24 (49.0%)	108 (51.4%)		23 (47.9%)	21 (43.8%)	
** 3 (T > 50)**	11 (22.4%)	60 (28.6%)		11 (22.9%)	13 (27.1%)	
** 4 (inflammatory BC)**	8 (16.3%)	21 (10.0%)		8 (16.7%)	8 (16.7%)	
**N stage**			**.104**			**.487**
** 0**	2 (4.1%)	5 (2.4%)		2 (4.2%)	0 (0.0%)	
** 1**	7 (14.3%)	62 (29.5%)		7 (14.6%)	10 (20.8%)	
** 2**	22 (44.9%)	71 (33.8%)		21 (43.8%)	18 (37.5%)	
** 3**	18 (36.7%)	72 (34.3%)		18 (37.5%)	20 (41.7%)	
**Stage**			**.110**			**.840**
** I A**	1 (2.0%)	0 (0.0%)		1 (2.1%)	0 (0.0%)	
** II A**	2 (4.1%)	9 (4.3%)		2 (4.2%)	2 (4.2%)	
** II B**	3 (6.1%)	37 (17.6%)		3 (6.3%)	4 (8.3%)	
** III A**	20 (40.8%)	80 (38.1%)		19 (39.6%)	20 (41.7%)	
** III B**	5 (10.2%)	12 (5.7%)		5 (10.4%)	2 (4.2%)	
** III C**	18 (36.7%)	72 (34.3%)		18 (37.5%)	20 (41.7%)	
**Ki67 level at baseline (%)**			**.056**			**1.000**
** <15**	12 (24.5%)	24 (11.4%)		12 (25%)	12 (25%)	
** 15-30**	21 (42.9%)	99 (47.1%)		21 (43.8%)	20 (41.7%)	
** >30**	16 (32.7%)	87 (41.4%)		15 (31.3%)	16 (33.3%)	
**ER level at baseline (%)**			**.119**			**.562**
** ≤50**	1 (2.0%)	19 (9.0%)		1 (2.1%)	1 (2.1%)	
** 51-80**	14 (28.6%)	74 (35.2%)		13 (27.1%)	8 (16.7%)	
** ≥81**	34 (69.4%)	117 (55.7%)		34 (70.8%)	39 (81.3%)	
**HER2 status at baseline**			**.228**			**.635**
** 0**	10 (20.4%)	46 (21.9%)		10 (20.8%)	7 (14.6%)	
** 1+**	23 (46.9%)	72 (34.3%)		23 (47.9%)	22 (45.8%)	
** 2+FISH-**	16 (32.7%)	92 (43.8%)		15 (31.3%)	19 (39.6%)	

Abbreviations: BC, breast cancer; ER, estrogen receptor; HER2, human epidermal growth factor receptor 2; IDC, invasive ductal carcinoma; NA, not available; PSM, propensity score matching. *p* values are shown in bold.

### The efficacy analysis of neoadjuvant endocrine therapy and neoadjuvant chemotherapy in HR+/HER2- breast cancer patients

**Figure 1.**
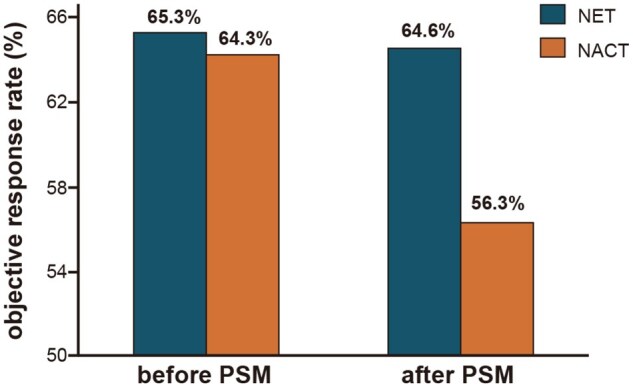
ORR of neoadjuvant treatment before and after PSM. NACT, neoadjuvant chemotherapy; NET, neoadjuvant endocrine therapy; ORR, objective response rate; PSM, propensity score matching.

According to clinical imaging evaluation, before PSM, the objective response rate (ORR) was 65.3% (95% CI, 50.4-78.3) in endocrine group and 64.3% (95% CI, 57.4-70.8) in chemotherapy group, with no statistically significant difference (*P *= .893) between them. Similarly, the clinical benefit rate (CBR) was 98.0% in endocrine group and 99.0% in chemotherapy group (*P *= .468, Table 2). After PSM, the ORR was 64.6% (95% CI, 49.5-77.8) in endocrine group compared to 56.3% (95% CI, 41.2-70.5) in chemotherapy group, remained statistically non-significant (*P *= .532, Figure 1). Respectively, the subgroup analysis also showed no statistically significant differences in ORR between endocrine group and chemotherapy group. After PSM, the ORR was slightly higher in endocrine group that for patients younger than 45 years (84.6% vs 61.5%, HR = 3.438, 95% CI, 0.527-22.432, *P *= .378), premenopausal women (77.8% vs 66.7%, HR = 1.750, 95% CI, 0.398-7.700, *P *= .711), non-invasive ductal carcinoma (IDC, 80.0% vs 50%, HR = 4.000, 95% CI, 0.211-75.659, *P *= .524), earlier N stage (66.7% vs 50%, HR = 2.000, 95% CI, 0.224-17.894, *P *= .627), lower Ki67 level (75.0% vs 41.7%, HR = 4.200, 95% CI, 0.738-23.907, *P *= .214), ER expression >80% (61.8% vs 51.3%, HR = 1.535, 95% CI, 0.603-3.906, *P *= .479). Conversely, the ORR was higher in chemotherapy group that for patients with late menarche (33.3% vs 50.0%, HR = 0.500, 95% CI, 0.084-2.992, *P *= .660), earlier T stage (25.0% vs 60.0%, HR = 0.222, 95% CI, 0.012-3.979, *P *= .524), and lower HER2 expression level (50.0% vs 77.8%, HR = 0.286, 95% CI, 0.035-2.322, *P *= .335; Figure S2, See online supplementary material for a color version of this figure). Figure S3 (See online supplementary material for a color version of this figure) showed the ORR of different neoadjuvant treatments before PSM, demonstrating a trend generally consistent with the ORR observed after PSM.

**Table 2.** Efficacy by neoadjuvant treatment before and after PSM.

	Before PSM			After PSM		
	Neoadjuvant endocrine therapy (*n* = 49)	Neoadjuvant chemotherapy (*n* = 210)	*P*	Neoadjuvant endocrine therapy (*n* = 48)	Neoadjuvant chemotherapy (*n* = 48)	*P*
**Best clinical overall response per RECIST v1.1, *n* (%)**						
** CR**	0 (0.0%)	1 (0.5%)		0 (0.0%)	0 (0.0%)	
** PR**	32 (65.3%)	134 (63.8%)		31 (64.6%)	27 (56.3%)	
** SD**	16 (32.7%)	73 (34.8%)		16 (33.3%)	20 (41.7%)	
** PD**	1 (2.0%)	2 (1.0%)		1 (2.1%)	1 (2.1%)	
** ORR, % (95% CI)**	65.3 (50.4-78.3)	64.3 (57.4-70.8)	**.893**	64.6 (49.5-77.8)	56.3 (41.2-70.5)	**.532**
** CBR, % (95% CI)**	98.0 (89.1-99.9)	99.0 (96.6-99.9)	**.468**	97.9 (88.9-99.9)	97.9 (88.9-99.9)	**1.000**

Abbreviations: CBR, clinical benefit rate; CI, confidence interval; CR, complete response; ORR, objective response rate; PD, partial disease; PR, partial response; PSM, propensity score matching; SD, stable disease. *p* values are shown in bold.

### Neoadjuvant endocrine therapy was associated with greater downregulation of Ki67 and ER/PR expression and improved Miller-Payne grading at surgery

**Figure 2.**
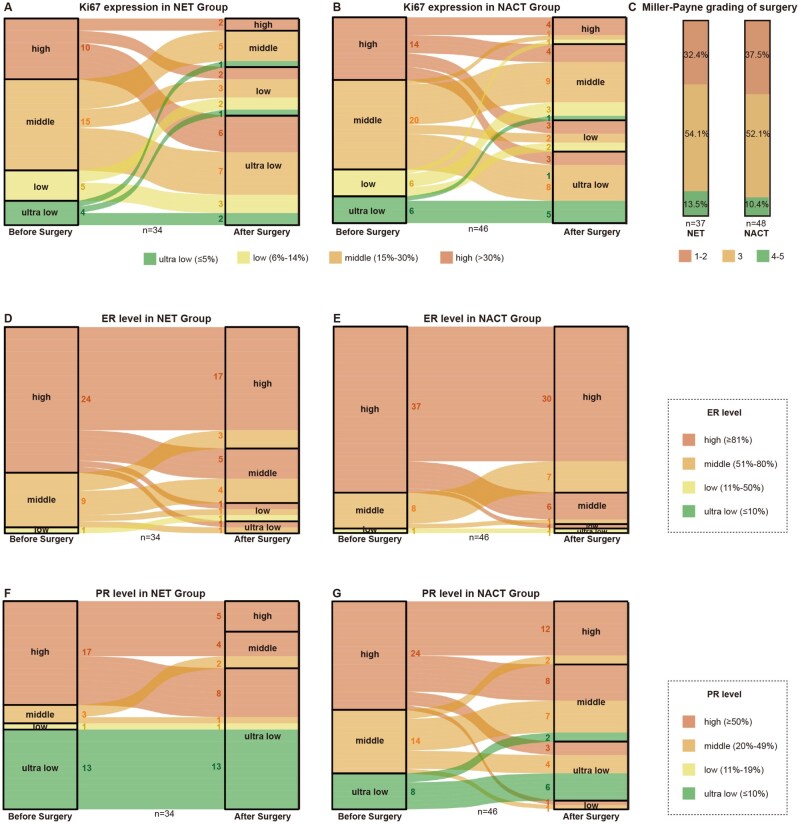
NET resulted in a greater downregulation of Ki67 and ER/PR expression and improved MP grading at surgery after PSM. A. Ki67 expression in NET group before and after surgery. B. Ki67 expression in NACT group before and after surgery. C. Miller-Payne grading of surgery of NET group and NACT group. D. ER level in NET group before and after surgery. E. ER level in NACT group before and after surgery. F. PR level NET group before and after surgery. G. PR level in NACT group before and after surgery. ER, estrogen receptor; NACT, neoadjuvant chemotherapy; NET, neoadjuvant endocrine therapy; PR, progesterone receptor; PSM, propensity score matching.

Previous studies have indicated that breast cancer patients with high baseline Ki67 levels were associated with a higher risk of recurrence and poorer prognosis. Furthermore, a high level of Ki67 expression in residual disease following neoadjuvant treatment has been correlated with unfavorable long-term outcomes.^15^^,^^16^ In this study, after PSM, 37 out of 48 patients in endocrine therapy group and all 48 patients in chemotherapy group underwent surgery. Changes in Ki67 expression were analyzed in patients who did not achieve a pathological complete response (pCR), defined as the absence of residual invasive disease in the primary lesion. Ki67 expression was categorized into four levels—ultra-low, low, middle, and high—using cutoff values of 5%, 15%, and 30%. As shown in Figure 2A and B, before surgery, the distribution of four Ki67 categories was generally similar between the two groups. However, endocrine group showed a significant post-surgical increase in the proportion of patients with ultra-low as well as low level of Ki67 expression, primarily due to a shift from the high and middle categories, particularly when compared to chemotherapy group. Notably, after neoadjuvant endocrine therapy, more than half of the patients who underwent surgery had Ki67 levels below 5%.

Miller-Payne grading is an independent predictor of DFS and OS.^17^ In this study, endocrine therapy group also demonstrated a stronger tumor-degrading effect, presented as a lower proportion of patients with Miller-Payne grade 1-2 (32.4% vs 37.5%) and a relatively higher proportion with grade 4-5 (13.5% vs 10.4%) compared to the chemotherapy group after PSM. This suggested that neoadjuvant endocrine therapy may result in a higher likelihood of achieving pCR or near-pCR (Figure 2C).

Among post-surgical patients, neoadjuvant endocrine therapy led to a greater reduction in tumors’ HR level. ER expression was categorized into four levels based on cut-off values of 10%, 50%, and 80%. PR expression was classified using thresholds of 10%, 20%, and 50%. As shown in Figure 2D and E, ER level generally decreased in endocrine group after surgery, whereas chemotherapy group maintained high ER expression, primarily due to a part of middle category shift to the high after PSM. Meanwhile, the proportion of ultra-low PR expression increased markedly following endocrine therapy (Figure 2F and G), suggesting that low PR levels may serve as a potential predictor of response to endocrine treatment. Figure S4 (See online supplementary material for a color version of this figure) illustrated the changes in Ki67 and ER/PR level, as well as Miller-Payne grading before PSM, which were generally consistent with the trends described above.

### Exploration of neoadjuvant endocrine therapy for tumors less sensitive to traditional neoadjuvant chemotherapy

According to the analysis above, nearly 40% of patients failed to achieve a partial response (PR) with traditional neoadjuvant chemotherapy. For both cohorts of this study during chemotherapy stage, 35 patients in endocrine therapy group and 119 patients in chemotherapy group did not achieve PR at the initial clinical imaging evaluation, typically conducted after 2-3 cycles of chemotherapy. Among this chemotherapy-insensitive subgroup, significant differences (*P *< .05) were observed between the 2 groups at the baseline characteristics in age at first diagnose (*P *= .023), N stage (*P *= .038), and overall clinical stage (*P *= .038) (Table S2). To reduce bias, a second PSM (PSM2) was conducted at a 1:1 ratio, resulting in a matched cohort of 68 patients. After matching, no statistically significant differences (*P *> .05) were observed in baseline characteristics between the two groups. Effect of PSM2 was illustrated in Figure S5 (See online supplementary material for a color version of this figure), and the matched patient list was provided in Table S3.

**Figure 3.**
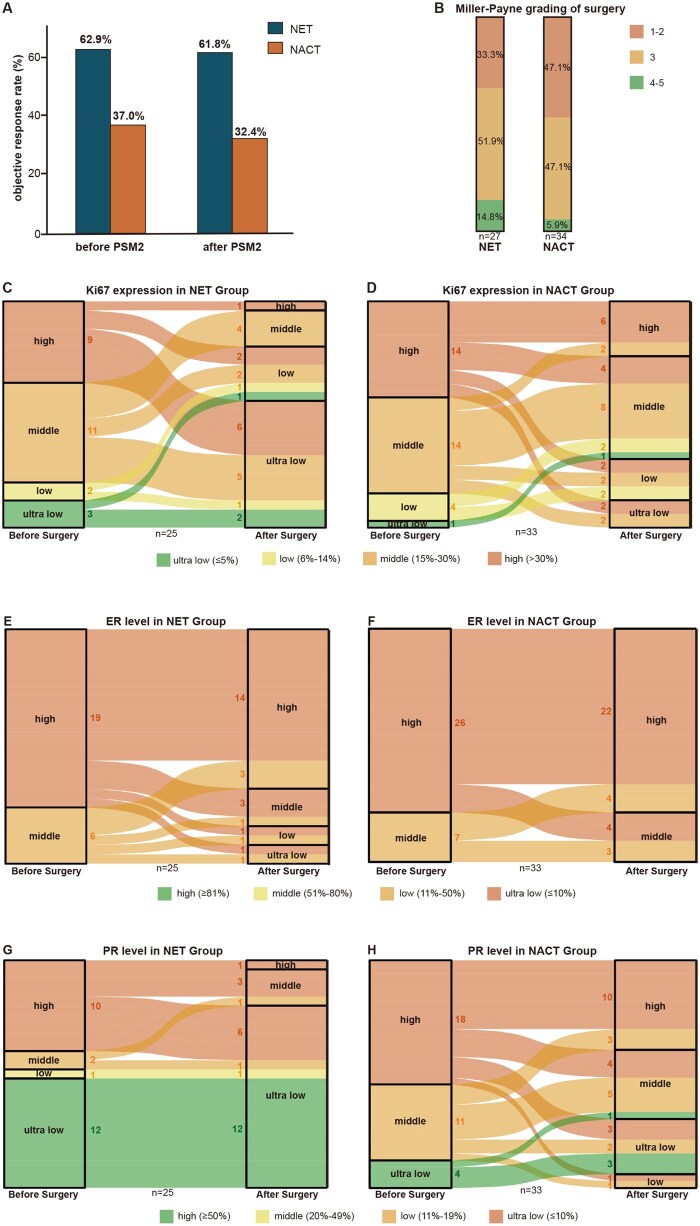
Populations less sensitive to NACT still benefit from ET analyzed after PSM2. A. ORR of neoadjuvant treatment before and after PSM2. B. MP grading of surgery of NET group and NACT group. C. Ki67 expression in NET group before and after surgery. D. Ki67 expression in NACT group before and after surgery. E. ER level in NET group before and after surgery. F. ER level in NACT group before and after surgery. G. PR level NET group before and after surgery. H. PR level in NACT group before and after surgery. ER, estrogen receptor; NACT, neoadjuvant chemotherapy; NET, neoadjuvant endocrine therapy; ORR, objective response rate; PR, progesterone receptor; PSM, propensity score matching.

Efficacy outcomes in populations less responsive to chemotherapy were evaluated. Before PSM2, the ORR was 62.9% (95% CI, 46.0-79.7) in endocrine group and 37.0% (95% CI, 28.2-45.8) in chemotherapy group, with a statistically significant difference (*P *= .011) between them (Table 3). After PSM2, the ORR remained significantly higher in endocrine group (61.8%, 95% CI, 44.6-79.0) compared to chemotherapy group (32.4%, 95% CI, 15.8-48.9) (*P *= .028, Figure 3A). These findings suggested that for patients who did not achieve PR at the initial evaluation following traditional neoadjuvant chemotherapy, subsequent treatment with endocrine therapy may lead to improved ORR.

**Table 3.** Efficacy outcomes in populations less responsive to neoadjuvant chemotherapy.

	Before PSM2			After PSM2		
	Neoadjuvant endocrine therapy (*n* = 35)	Neoadjuvant chemotherapy (*n* = 119)	*P*	Neoadjuvant endocrine therapy (*n* = 34)	Neoadjuvant chemotherapy (*n* = 34)	*P*
**Best clinical overall response per RECIST v1.1, *n* (%)**						
** CR**	0 (0.0%)	0 (0.0%)		0 (0.0%)	0 (0.0%)	
** PR**	22 (62.9%)	44 (37.0%)		21 (61.8%)	11 (32.4%)	
** SD**	12 (34.3%)	73 (61.3%)		12 (35.3%)	22 (64.7%)	
** PD**	1 (2.9%)	2 (1.7%)		1 (2.9%)	1 (2.9%)	
** ORR, % (95% CI)**	62.9 (46.0-79.7)	37.0 (28.2-45.8)	**.011**	61.8 (44.6-79.0)	32.4 (15.8-48.9)	**.028**
** CBR, % (95% CI)**	97.1 (91.3-102.9)	98.3 (96.0-100.7)	.541	97.1 (91.1-103.0)	97.1 (91.1-103.0)	1.000

Abbreviations: CBR, clinical benefit rate; CI, confidence interval; CR, complete response; ORR, objective response rate; PD, partial disease; PR, partial response; PSM, propensity score matching; SD, stable disease. *p* values are shown in bold.

After PSM2, 27 out of 34 patients in endocrine group and all 34 patients in chemotherapy group underwent surgery. Endocrine group demonstrated a stronger tumor-degrading effect showed as a significantly lower proportion of patients with Miller-Payne grade 1-2 (33.3% vs 47.1%) and a higher proportion with grade 4-5 (14.8% vs 5.9%) compared to the chemotherapy group, which indicated a greater likelihood of achieving pCR or near-pCR, even surpassing the outcomes observed in the first PSM analysis (Figure 3B). And changes in Ki67 expression were analyzed among patients who did not achieve pCR. As shown in Figure 3C and D, endocrine group exhibited a significant post-surgical increase in the proportion of patients with ultra-low and low Ki67 expression. Following neoadjuvant endocrine therapy, the majority of surgical patients had Ki67 level below 5%. Neoadjuvant endocrine therapy also led to a decrease in HR level. As illustrated in Figure 3E and F, ER level declined overall in endocrine group after surgery, whereas chemotherapy group maintained high ER expression. Furthermore, the proportion of patients with ultra-low PR expression markedly increased following endocrine therapy (Figure 3G and H). Figure S6 (See online supplementary material for a color version of this figure) presented the changes in Ki67 and ER/PR level, as well as Miller-Payne grading before PSM2, which were generally consistent with the trends described above.

**Figure 4.**
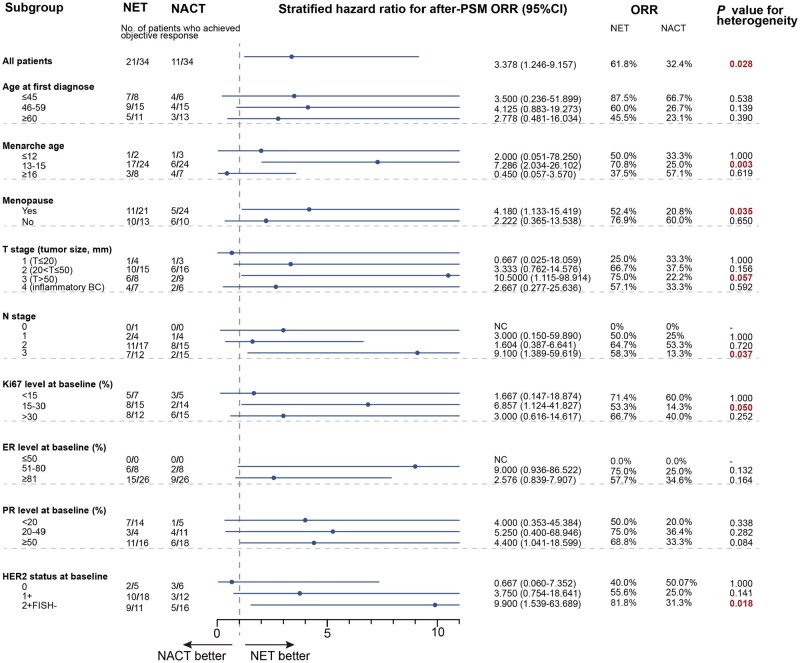
ORR of neoadjuvant treatment in subgroups after PSM2. ER, estrogen receptor; HER2: human epidermal growth factor receptor 2; NACT, neoadjuvant chemotherapy; NET, neoadjuvant endocrine therapy; ORR, objective response rate; PR, progesterone receptor; PSM, propensity score matching.

Subgroup analysis revealed a general advantage of neoadjuvant endocrine therapy over chemotherapy in tumors that were less sensitive to traditional chemotherapy, with several subgroups showing statistically significant differences. As shown in Figure 4, after PSM2, endocrine group demonstrated a significantly higher ORR among patients who were postmenopausal (52.4% vs 20.8%, HR = 4.180, 95% CI, 1.133-15.419, *P *= .035), T stage 3 disease (75.0 vs 22.2%, HR = 10.500, 95% CI, 1.115-98.914, *P *= .057) or N stage 3 disease (58.3% vs 13.3%, HR = 9.100, 95% CI, 1.389-59.619, *P *= .037), Ki67 expression between 15% and 30% (53.3% vs 14.3%, HR = 6.857, 95% CI, 1.124-41.827, *P *= .050), or higher level of HER2 expression (81.8% vs 31.3%, HR = 9.900, 95% CI, 1.539-63.689, *P *= .018). These findings may help identify populations more likely to benefit from neoadjuvant endocrine therapy. Figure S7 (See online supplementary material for a color version of this figure) presented the results of subgroup analysis before PSM2, which showed a generally similar trend to analysis above, though some subgroup differences were observed.

## Discussion

In this study, we conducted a retrospective cohort analysis of breast cancer patients who received neoadjuvant endocrine therapy combined with CDK4/6 inhibitors. By comparing their treatment outcomes with those who underwent traditional neoadjuvant chemotherapy, we aimed to explore if there might have broader applications of endocrine therapy in HR-positive breast cancer and to identify potential populations that may derive greater benefit from this approach. Our results showed that across two cohorts of neoadjuvant strategies, the ORR was statistically non-significant between neoadjuvant endocrine therapy and traditional chemotherapy (64.6 vs 56.3, *P *= .532; Table 2, Figure 1), and the near-pCR rate (Miller-Payne grade 4-5) was 13.4% in endocrine therapy group vs 10.5% in chemotherapy group (Figure 2C). These findings suggested that the overall efficacy of neoadjuvant endocrine therapy might not be inferior to traditional neoadjuvant chemotherapy. The previous ADAPTcycle study also compared neoadjuvant endocrine therapy plus CDK4/6 inhibitor with neoadjuvant chemotherapy and reported an AI response rate exceeding 80%, significantly higher than tamoxifen, with response defined as Ki67 ≤ 10% after endocrine therapy. In that study, the pCR rate in ribociclib plus endocrine group was 5.7%, comparable to 7.1% in chemotherapy group (*P *= .542). Subgroup analysis further showed that endocrine therapy responders had a higher pCR rate than non-responders.^13^ These findings were consistent with our own. Although overall response rates to neoadjuvant endocrine therapy still need improvement and no statistically superior outcomes over neoadjuvant chemotherapy have been observed thus far, identifying suitable patient subgroups may lead to breakthroughs.

Subgroup analysis in this study tried to provide an initial insight into populations that are more likely to benefit from neoadjuvant endocrine therapy. The subgroups with higher ORR include younger or premenopausal women, patients with earlier N stage, lower baseline Ki67, and higher ER/PR or HER2 expression, although no statistical differences were observed (Figure S2—See online supplementary material for a color version of this figure). Among them, younger and premenopausal patients, as well as those with high ER/PR expression, may respond better to endocrine therapy due to relatively higher estrogen level.^18^ For Ki67, prior trials, such as P024^19^ and IMPACT^20^ found that patients with low baseline Ki67 (<10%) had excellent 5-years survival, and assessing Ki67 after 2-4 weeks of neoadjuvant endocrine therapy is a valuable biomarker for treatment efficacy in patients with baseline Ki67 > 10%.^10^ Interestingly, HER2 status also emerged as a relevant factor. Patients with higher HER2 expression appeared to benefit more from neoadjuvant endocrine therapy (Figures S2, S3, and S7—See online supplementary material for a color version of this figure; Figure 4). This aligns with findings from the PALOMA-2 and PALOMA-3 trials, which showed HER2-low patients experienced greater benefit from endocrine therapy combined with CDK4/6 inhibitors than HER2-0 patients, though these studies were conducted in metastatic breast cancer.^21^ The underlying mechanism may involve ER-HER2 signaling pathways crosstalk contributing to endocrine therapy resistance in HER2-low tumors,^22^ which can be overcome by CDK4/6 inhibitors through interruption of HER2 and its downstream pathways, such as PI3K/AKT/mTOR and MAPK.^23^ Further, more evidence is still needed to precise patient selection that optimize the use of neoadjuvant endocrine therapy in HR-positive breast cancer.

Furthermore, this study explored a simple and practical neoadjuvant mode: tailoring subsequent therapy based on response to initial induction chemotherapy. Our analysis showed that among patients who failed to achieve PR after 2-3 cycles of neoadjuvant chemotherapy, only 37% eventually responded by the end of treatment. For these chemotherapy-insensitive tumors, switching to neoadjuvant endocrine therapy may offer a promising alternative (Table 3, Figure 2A). Several studies have investigated neoadjuvant endocrine therapy in HR-positive breast cancer. Trials such as neoMONARCH and CORALLEEN have shown that AIs combined with CDK4/6 inhibitors before surgery can achieve outcomes not inferior to neoadjuvant chemotherapy.^24^^,^^25^ Specifically, the FINEST study that with the mode of screening population that suitable for neoadjuvant endocrine therapy based on chemotherapy sensitivity found that switching to endocrine-immunotherapy improved ORR in chemotherapy-insensitive subgroup, though it did not enhance pCR rate.^26^ While our study observed that transitioning chemotherapy-insensitive subgroup to neoadjuvant endocrine therapy plus CDK4/6 inhibitors led to improved ORR, as well as higher near-pCR rates and greater Ki67 suppression (Figure 3A-D), suggesting a potential therapeutic advantage. Although preliminary, these findings provide encouragement for further exploration of this mode.

Therapeutic regimen selection is another important consideration. In our cohort, patients treated with SERD-regulators (eg, fulvestrant) combined with CDK4/6 inhibitors (palbociclib, dalciclib, or abemaciclib) achieved an ORR of 85.7%, notably higher than the 61.0% observed with AIs plus CDK4/6 inhibitors. Various neoadjuvant endocrine therapy strategies continue to be investigated, including SERMs plus ovarian function suppression (OFS),^27^ AIs alone (eg, Alliance Z1031 study),^28^ SERD ± CDK4/6 inhibitors,^29^ and novel combinations, such as T-DM1 ± endocrine therapy for HR+/HER2+ patients (WSG-ADAPT-TP Trial).^30^ However, more robust clinical data are needed to fully evaluate their efficacy and safety.

This study had several limitations. It was a retrospective, single-center cohort study with multiple treatment regimens and variable strategies. Future prospective randomized controlled trials (RCT) focusing on chemotherapy-insensitive populations after four cycles of chemotherapy are expected to conduct.

## Conclusion

Neoadjuvant endocrine therapy combined with CDK4/6 inhibitors is a promising alternative for HR-positive breast cancer, especially for patients with poor response to chemotherapy.

## Supplementary Material

oyag032_Supplementary_Data

## Data Availability

The datasets used and analysed during the current study are available from the corresponding author on reasonable request.
