# Supplementary material for: Efficacy of neoadjuvant endocrine therapy with CDK4/6 inhibitors in locally advanced breast cancer
Source: Oncologist. 2026 Feb 6;31(3):oyag032. doi: 10.1093/oncolo/oyag032 (PMC12952918; doi:10.1093/oncolo/oyag032)
Supplement: oyag032_Supplementary_Data [file oyag032_supplementary_data.zip › Supplementary Figure.docx]

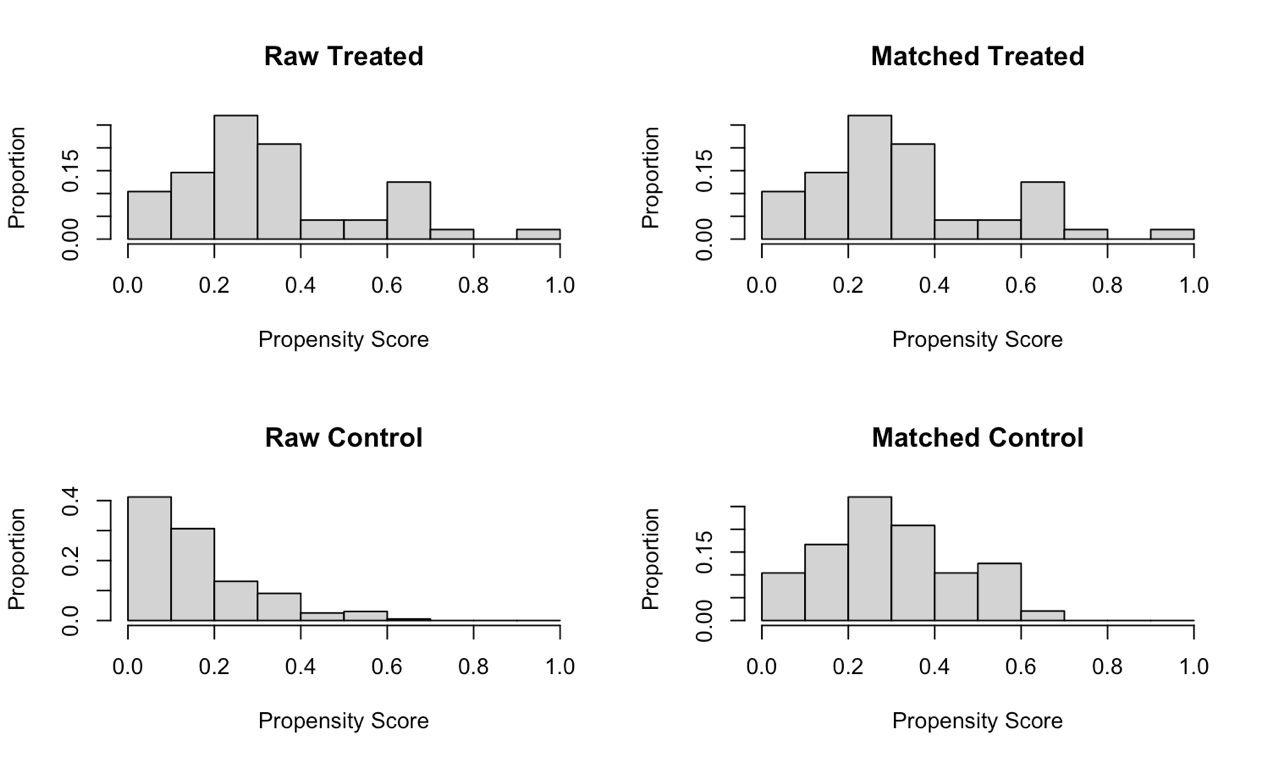


**Supplementary Figure 1.** Effect of PSM. Before PSM, there were significant differences in baseline characteristics between the treatment group (endocrine group) and the control group (chemotherapy group). After PSM, the groups were well balanced and comparable. **PSM:** propensity score matching.

**
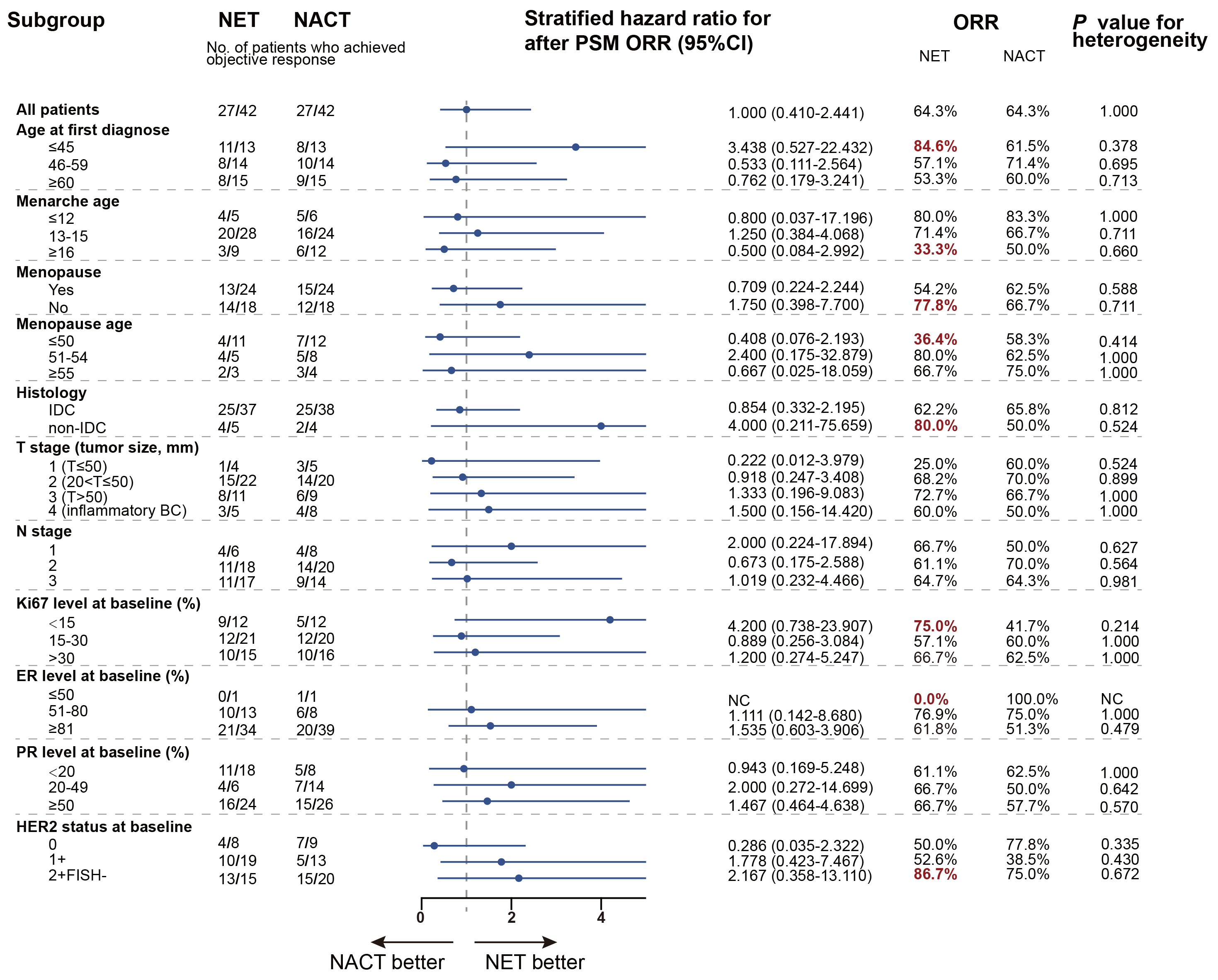
**

**Supplementary Figure 2.** ORR of neoadjuvant treatment in subgroups after PSM. **NET:** neoadjuvant endocrine therapy; **NACT:** neoadjuvant chemotherapy; **PSM:** propensity score matching; **ORR:** objective response rate; **ER:** estrogen receptor; **PR:** progesterone receptor; **HER2:** human epidermal growth factor receptor 2.


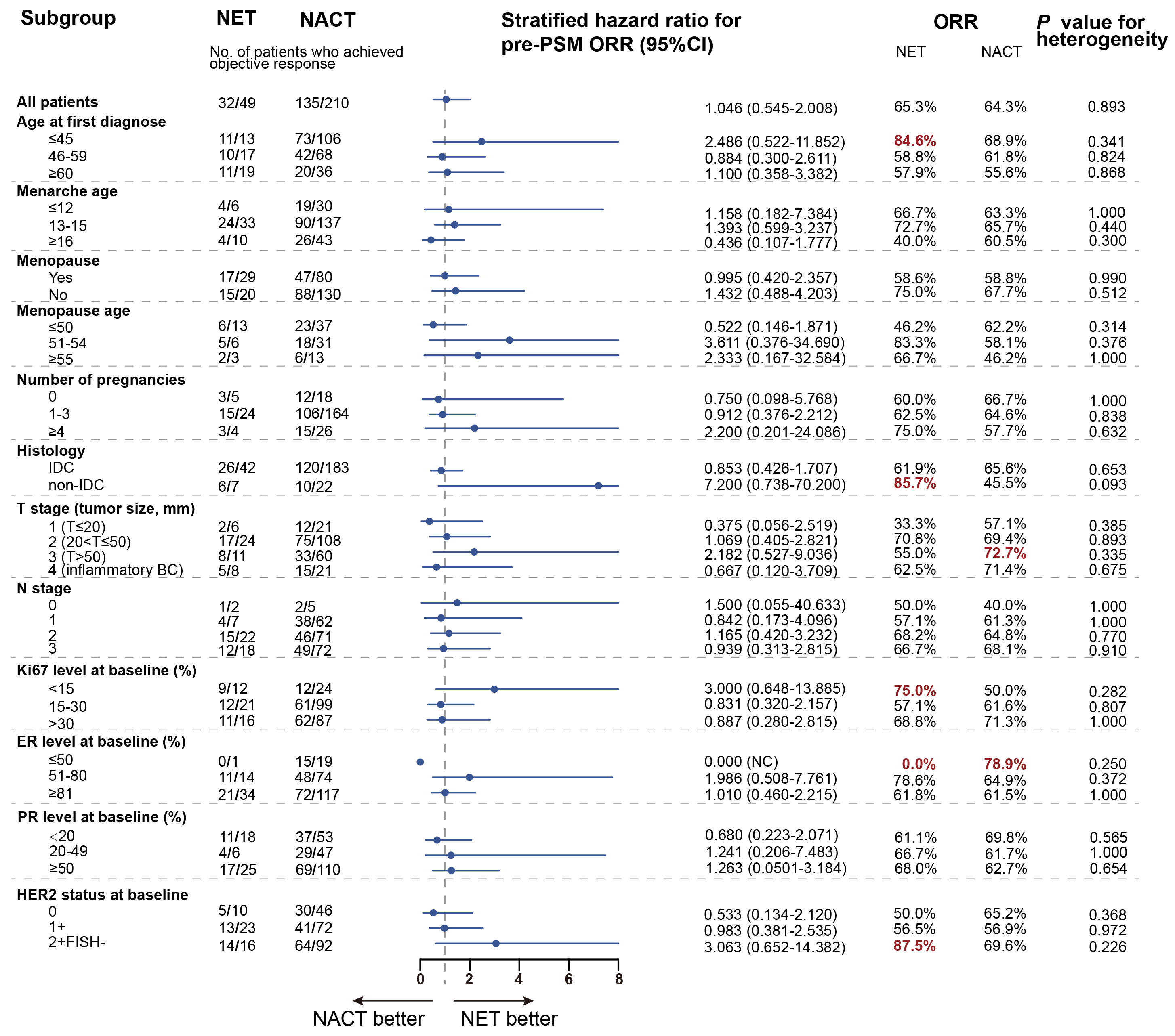


**Supplementary Figure 3.** ORR of neoadjuvant treatment in subgroups before PSM. **NET:** neoadjuvant endocrine therapy; **NACT:** neoadjuvant chemotherapy; **PSM:** propensity score matching; **ORR:** objective response rate; **ER:** estrogen receptor; **PR:** progesterone receptor; **HER2:** human epidermal growth factor receptor 2.


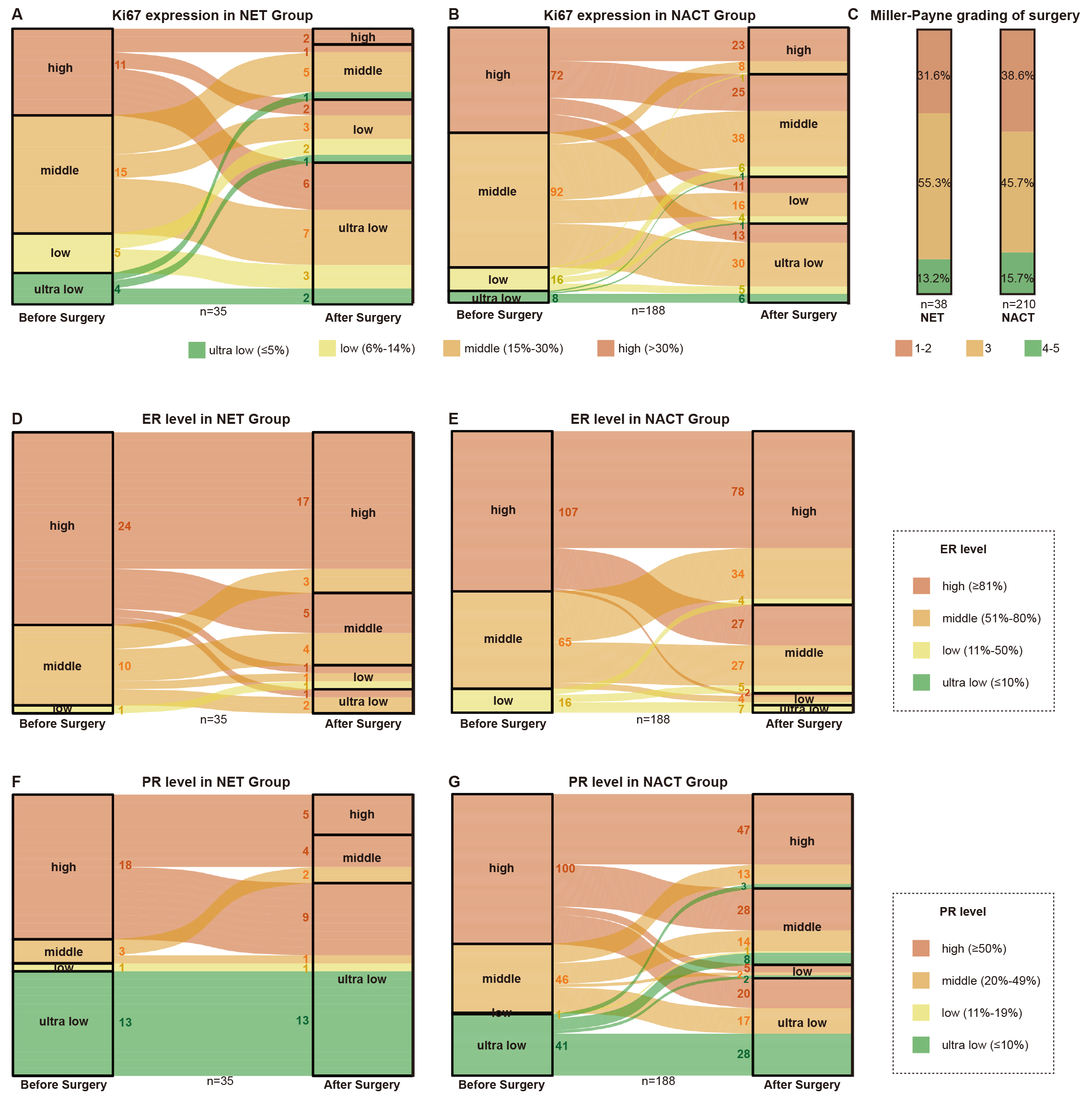


**Supplementary Figure 4.** NET resulted in a greater downregulation of Ki67 and ER/PR expression and improved MP grading at surgery before PSM. **A.** Ki67 expression in NET group before and after surgery. **B.** Ki67 expression in NACT group before and after surgery. **C.** Miller-Payne grading of surgery of NET group and NACT group. **D.** ER level in NET group before and after surgery. **E.** ER level in NACT group before and after surgery. **F.** PR level NET group before and after surgery. **G.** PR level in NACT group before and after surgery. **NET:** neoadjuvant endocrine therapy; **NACT:** neoadjuvant chemotherapy; **PSM:** propensity score matching; **ER:** estrogen receptor; **PR:** progesterone receptor.


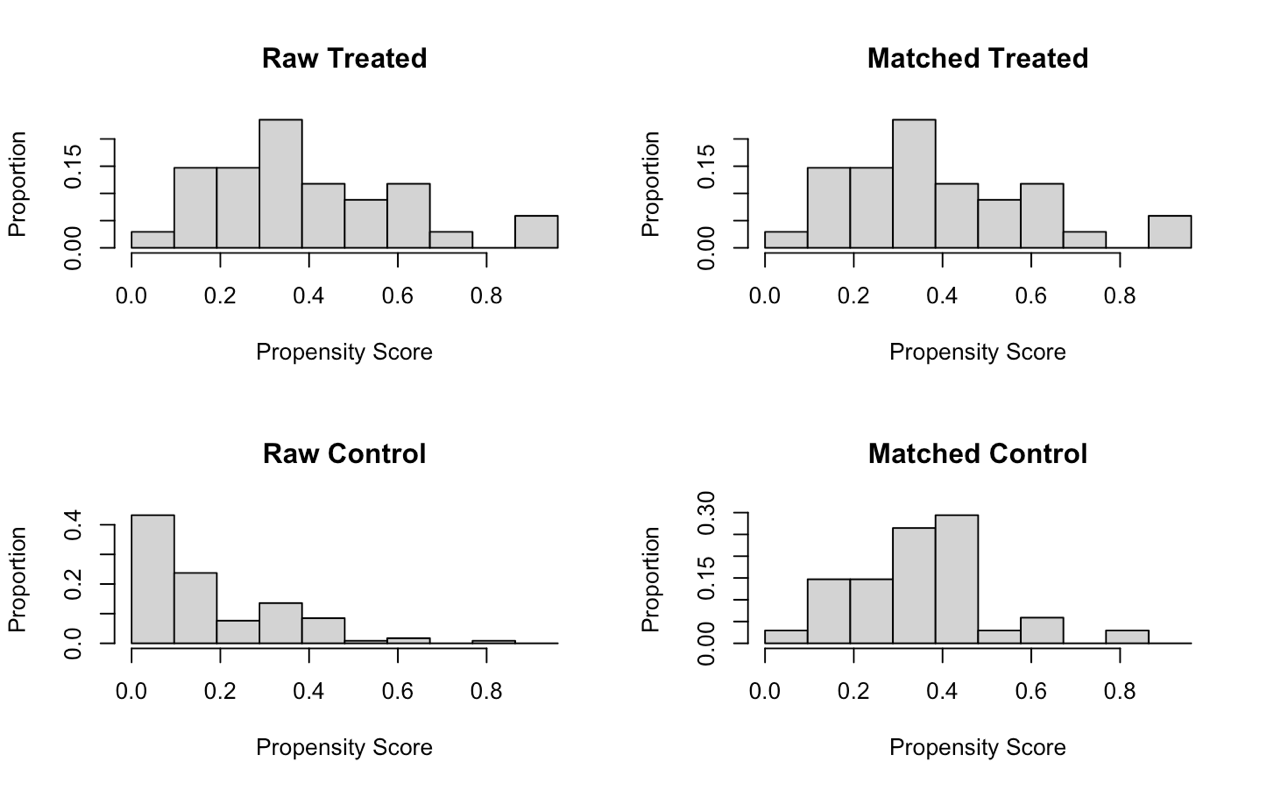


**Supplementary Figure 5.** Effect of PSM2. Before PSM2, there were significant differences in baseline characteristics between the treatment group (endocrine group) and the control group (chemotherapy group). After PSM2, the groups were well balanced and comparable. **PSM:** propensity score matching.


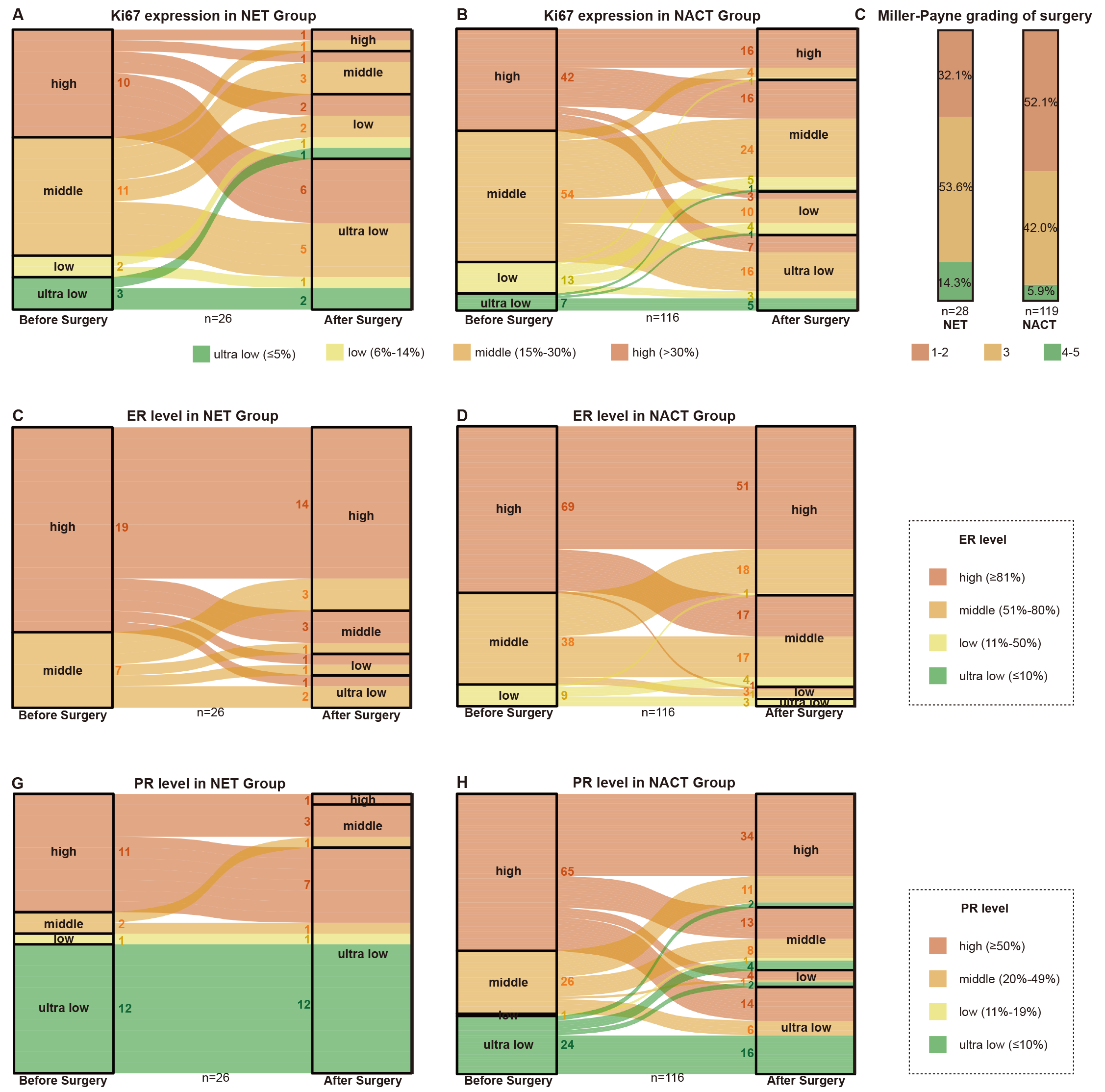


**Supplementary Figure 6.** NET resulted in a greater downregulation of Ki67 and ER/PR expression and improved MP grading at surgery before PSM2. **A.** Ki67 expression in NET group before and after surgery. **B.** Ki67 expression in NACT group before and after surgery. **C.** Miller-Payne grading of surgery of NET group and NACT group. **D.** ER level in NET group before and after surgery. **E.** ER level in NACT group before and after surgery. **F.** PR level NET group before and after surgery. **G.** PR level in NACT group before and after surgery. **NET:** neoadjuvant endocrine therapy; **NACT:** neoadjuvant chemotherapy; **PSM:** propensity score matching; **ER:** estrogen receptor; **PR:** progesterone receptor.


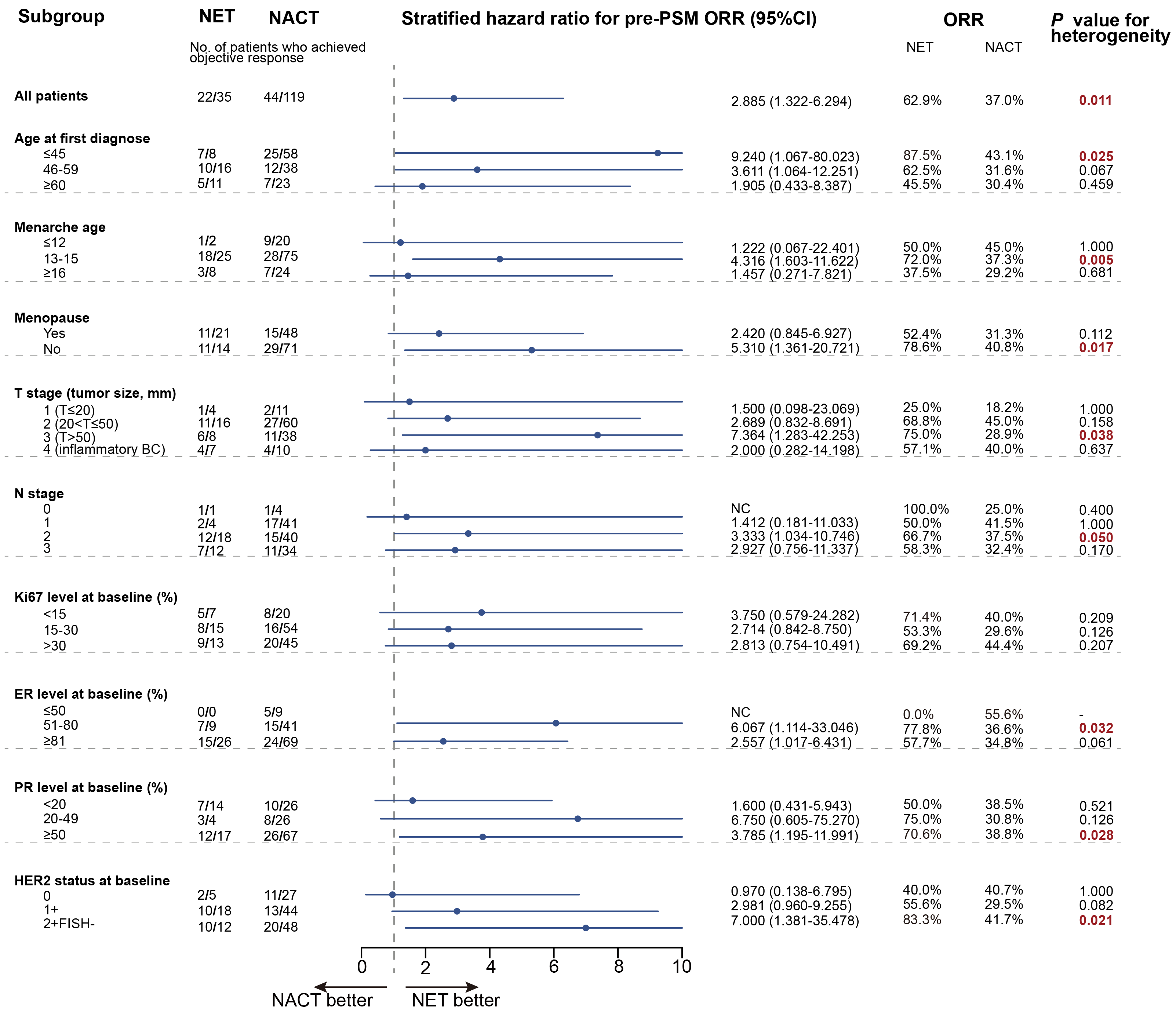


**Supplementary Figure 7.** ORR of neoadjuvant treatment in subgroups before PSM2. **NET:** neoadjuvant endocrine therapy; **NACT:** neoadjuvant chemotherapy; **PSM:** propensity score matching; **ORR:** objective response rate; **ER:** estrogen receptor; **PR:** progesterone receptor; **HER2:** human epidermal growth factor receptor 2.
