# Supplementary material for: Efficacy of neoadjuvant endocrine therapy with CDK4/6 inhibitors in locally advanced breast cancer
Source: Oncologist. 2026 Feb 6;31(3):oyag032. doi: 10.1093/oncolo/oyag032 (PMC12952918; doi:10.1093/oncolo/oyag032)
Supplement: oyag032_Supplementary_Data [file oyag032_supplementary_data.zip › Supplementary Table.docx]

**Supplementary Table 1.** PSM list

|  | **treatment** | **ID** | **age** | **menarche**  **age** | **menopause** | **type** | **grade** | **T** | **N** | **stage** | **Ki67** | **ER** | **HER2** | **distance** | **weights** | **subclass** |
| --- | --- | --- | --- | --- | --- | --- | --- | --- | --- | --- | --- | --- | --- | --- | --- | --- |
| 1 | 1 | 1854110 | 2 | 2 | 1 | IDC | median | 2 | 2 | IIIA | 1 | 2 | 0 | 0.357227 | 1 | 1 |
| 2 | 1 | 1887046 | 3 | 2 | 1 | IDC | median | 2 | 3 | IIIC | 2 | 2 | 0 | 0.313258 | 1 | 2 |
| 3 | 1 | 1056883 | 3 | 2 | 1 | non-IDC | high | 4 | 2 | IIIB | 1 | 3 | 1 | 0.921715 | 1 | 3 |
| 4 | 1 | 1E+09 | 3 | 2 | 1 | IDC | median | 4 | 1 | IIIB | 2 | 3 | 1 | 0.62438 | 1 | 4 |
| 5 | 1 | 1926609 | 2 | 2 | 1 | IDC | median | 4 | 2 | IIIB | 2 | 3 | 1 | 0.638318 | 1 | 5 |
| 6 | 1 | 1857151 | 1 | 2 | 0 | IDC | median | 2 | 3 | IIIC | 2 | 3 | 1 | 0.270073 | 1 | 6 |
| 7 | 1 | 1879154 | 2 | 2 | 1 | IDC | median | 2 | 0 | IIA | 2 | 3 | 1 | 0.338736 | 1 | 7 |
| 8 | 1 | 1906651 | 3 | 2 | 1 | IDC | median | 3 | 2 | IIIA | 3 | 3 | 1 | 0.617168 | 1 | 8 |
| 9 | 1 | 1921661 | 2 | 2 | 1 | non-IDC | median | 3 | 2 | IIIA | 3 | 3 | 1 | 0.402615 | 1 | 9 |
| 10 | 1 | 1926429 | 3 | 2 | 1 | IDC | median | 2 | 2 | IIIA | 2 | 3 | 2 | 0.322594 | 1 | 10 |
| 11 | 1 | 1974922 | 2 | 3 | 1 | IDC | median | 1 | 3 | IIIC | 2 | 3 | 0 | 0.201378 | 1 | 11 |
| 12 | 1 | 1957391 | 1 | 2 | 0 | IDC | median | 2 | 2 | IIIA | 3 | 2 | 1 | 0.226562 | 1 | 12 |
| 13 | 1 | 1964028 | 2 | 3 | 1 | IDC | median | 2 | 2 | IIIA | 2 | 2 | 1 | 0.234838 | 1 | 13 |
| 14 | 1 | 1969561 | 1 | 2 | 0 | IDC | median | 2 | 3 | IIIC | 1 | 2 | 1 | 0.323195 | 1 | 14 |
| 15 | 1 | 1732872 | 3 | 3 | 1 | IDC | median | 2 | 3 | IIIC | 1 | 3 | 0 | 0.693313 | 1 | 15 |
| 16 | 1 | 1780590 | 3 | 2 | 1 | non-IDC | median | 2 | 3 | IIIC | 2 | 3 | 0 | 0.487533 | 1 | 16 |
| 17 | 1 | 1778831 | 1 | 2 | 0 | IDC | median | 2 | 1 | IIB | 1 | 2 | 1 | 0.080727 | 1 | 17 |
| 18 | 1 | 1789815 | 3 | 3 | 1 | IDC | low | 4 | 3 | IIIC | 2 | 3 | 1 | 0.276164 | 1 | 18 |
| 19 | 1 | 1776627 | 1 | 3 | 0 | non-IDC | median | 3 | 3 | IIIC | 3 | 3 | 2 | 0.11423 | 1 | 19 |
| 20 | 1 | 1823375 | 1 | 1 | 0 | IDC | median | 3 | 2 | IIIA | 3 | 3 | 2 | 0.129379 | 1 | 20 |
| 21 | 1 | 1866867 | 2 | 2 | 0 | IDC | median | 4 | 2 | IIIB | 1 | 2 | 1 | 0.694915 | 1 | 21 |
| 22 | 1 | 1869032 | 3 | 1 | 1 | IDC | median | 4 | 3 | IIIC | 2 | 3 | 2 | 0.338936 | 1 | 22 |
| 23 | 1 | 1840635 | 2 | 3 | 1 | IDC | median | 2 | 2 | IIIA | 1 | 3 | 2 | 0.321153 | 1 | 23 |
| 24 | 1 | 1939921 | 2 | 3 | 0 | IDC | median | 2 | 2 | IIIA | 1 | 3 | 2 | 0.321153 | 1 | 24 |
| 25 | 1 | 1980918 | 3 | 2 | 1 | IDC | median | 2 | 3 | IIIC | 2 | 3 | 1 | 0.555244 | 1 | 25 |
| 26 | 1 | 1465138 | 3 | 2 | 1 | IDC | low | 2 | 2 | IIIA | 3 | 3 | 0 | 0.242973 | 1 | 26 |
| 27 | 1 | 1984042 | 1 | 2 | 0 | IDC | median | 3 | 2 | IIIA | 3 | 2 | 2 | 0.07049 | 1 | 27 |
| 28 | 1 | 1985960 | 3 | 2 | 1 | non-IDC | median | 4 | 3 | IIIC | 2 | 2 | 2 | 0.217816 | 1 | 28 |
| 29 | 1 | 2023393 | 1 | 2 | 0 | IDC | median | 3 | 2 | IIIA | 3 | 3 | 0 | 0.254894 | 1 | 29 |
| 30 | 1 | 2015365 | 1 | 1 | 0 | IDC | median | 1 | 2 | IIIA | 3 | 3 | 0 | 0.227896 | 1 | 30 |
| 31 | 1 | 1804964 | 3 | 2 | 1 | IDC | high | 1 | 2 | IIIA | 1 | 3 | 1 | 0.730266 | 1 | 31 |
| 32 | 1 | 2056243 | 2 | 2 | 1 | IDC | median | 2 | 2 | IIIA | 3 | 3 | 2 | 0.191346 | 1 | 32 |
| 33 | 1 | 2043250 | 3 | 2 | 1 | IDC | median | 3 | 2 | IIIA | 2 | 3 | 2 | 0.283864 | 1 | 33 |
| 34 | 1 | 2050977 | 1 | 1 | 0 | IDC | median | 2 | 2 | IIIA | 3 | 3 | 2 | 0.151489 | 1 | 34 |
| 35 | 0 | 2054954 | 2 | 2 | 0 | IDC | median | 2 | 2 | IIIA | 1 | 3 | 2 | 0.321153 | 1 | 23 |
| 36 | 0 | 2032074 | 1 | 2 | 0 | IDC | high | 2 | 3 | IIIC | 1 | 3 | 2 | 0.220785 | 1 | 12 |
| 37 | 0 | 2022016 | 2 | 1 | 1 | IDC | median | 3 | 3 | IIIC | 2 | 3 | 0 | 0.226155 | 1 | 30 |
| 38 | 0 | 2008852 | 2 | 2 | 1 | IDC | median | 2 | 2 | IIIA | 3 | 3 | 2 | 0.191346 | 1 | 32 |
| 39 | 0 | 1991407 | 3 | 2 | 1 | IDC | median | 2 | 3 | IIIC | 3 | 2 | 1 | 0.446258 | 1 | 25 |
| 40 | 0 | 1997414 | 3 | 1 | 1 | IDC | median | 4 | 3 | IIIC | 3 | 3 | 2 | 0.393412 | 1 | 9 |
| 41 | 0 | 1995232 | 1 | 2 | 0 | IDC | median | 2 | 2 | IIIA | 3 | 3 | 2 | 0.151489 | 1 | 34 |
| 42 | 1 | 1970683 | 2 | 2 | 1 | IDC | median | 3 | 3 | IIIC | 2 | 3 | 1 | 0.28986 | 1 | 35 |
| 43 | 0 | 1968526 | 3 | 2 | 1 | IDC | median | 1 | 1 | IIA | 1 | 3 | 1 | 0.518191 | 1 | 5 |
| 44 | 1 | 1866995 | 3 | 3 | 1 | IDC | median | 2 | 1 | IIB | 3 | 3 | 0 | 0.172139 | 1 | 36 |
| 45 | 0 | 1962356 | 3 | 2 | 1 | IDC | median | 3 | 2 | IIIA | 1 | 3 | 2 | 0.500621 | 1 | 4 |
| 46 | 1 | 1956358 | 1 | 2 | 0 | IDC | median | 3 | 1 | IIIA | 1 | 1 | 1 | 0.066586 | 1 | 37 |
| 47 | 1 | 1948704 | 3 | 2 | 1 | IDC | low | 1 | 3 | IIIC | 3 | 3 | 1 | 0.207918 | 1 | 38 |
| 48 | 0 | 1930626 | 3 | 2 | 1 | non-IDC | median | 3 | 3 | IIIC | 2 | 3 | 1 | 0.525158 | 1 | 21 |
| 49 | 1 | 1930502 | 3 | 2 | 1 | IDC | median | 2 | 3 | IIIC | 3 | 3 | 1 | 0.612285 | 1 | 39 |
| 50 | 0 | 1920042 | 3 | 3 | 1 | IDC | median | 2 | 2 | IIIA | 2 | 3 | 2 | 0.322594 | 1 | 10 |
| 51 | 0 | 1876462 | 2 | 2 | 1 | IDC | median | 4 | 3 | IIIC | 2 | 2 | 2 | 0.093196 | 1 | 41 |
| 52 | 0 | 1879383 | 3 | 3 | 1 | IDC | median | 1 | 3 | IIIC | 2 | 3 | 2 | 0.218055 | 1 | 28 |
| 53 | 0 | 1854810 | 3 | 2 | 1 | IDC | median | 4 | 3 | IIIC | 2 | 3 | 2 | 0.338936 | 1 | 22 |
| 54 | 1 | 1921024 | 2 | 2 | 0 | IDC | median | 2 | 3 | IIIC | 2 | 2 | 1 | 0.200159 | 1 | 40 |
| 55 | 1 | 1918277 | 2 | 2 | 0 | IDC | median | 4 | 1 | IIIB | 2 | 2 | 2 | 0.093904 | 1 | 41 |
| 56 | 1 | 1910117 | 2 | 2 | 0 | IDC | median | 3 | 2 | IIIA | 2 | 3 | 2 | 0.134723 | 1 | 42 |
| 57 | 0 | 1890346 | 2 | 2 | 1 | IDC | median | 2 | 2 | IIIA | 2 | 2 | 1 | 0.234838 | 1 | 13 |
| 58 | 0 | 1894118 | 1 | 2 | 0 | IDC | median | 4 | 1 | IIIB | 2 | 3 | 2 | 0.132869 | 1 | 42 |
| 59 | 0 | 1841706 | 1 | 2 | 0 | IDC | median | 3 | 2 | IIIA | 3 | 2 | 1 | 0.196026 | 1 | 40 |
| 60 | 0 | 1829291 | 2 | 3 | 0 | IDC | median | 2 | 2 | IIIA | 1 | 3 | 0 | 0.521314 | 1 | 15 |
| 61 | 0 | 1850082 | 3 | 2 | 1 | IDC | median | 1 | 2 | IIIA | 3 | 3 | 1 | 0.581757 | 1 | 3 |
| 62 | 1 | 1898553 | 1 | 2 | 0 | IDC | median | 2 | 3 | IIIC | 3 | 3 | 1 | 0.318819 | 1 | 43 |
| 63 | 1 | 884353 | 2 | 1 | 0 | IDC | median | 1 | 0 | IA | 2 | 2 | 0 | 1 | 1 | 44 |
| 64 | 1 | 2058269 | 1 | 1 | 0 | IDC | median | 2 | 1 | IIB | 2 | 3 | 1 | 0.063707 | 1 | 45 |
| 65 | 1 | 1961816 | 3 | 2 | 1 | non-IDC | high | 3 | 3 | IIIC | 1 | 2 | 2 | 0.301775 | 1 | 46 |
| 66 | 1 | 2012490 | 3 | 3 | 1 | IDC | median | 2 | 3 | IIIC | 2 | 3 | 1 | 0.555244 | 1 | 47 |
| 67 | 0 | 1949680 | 3 | 2 | 1 | IDC | median | 2 | 1 | IIB | 3 | 3 | 0 | 0.172139 | 1 | 36 |
| 68 | 0 | 1810435 | 1 | 2 | 0 | IDC | median | 3 | 1 | IIIA | 2 | 2 | 1 | 0.066564 | 1 | 37 |
| 69 | 0 | 1824782 | 1 | 2 | 0 | IDC | median | 3 | 3 | IIIC | 3 | 3 | 1 | 0.280355 | 1 | 33 |
| 70 | 0 | 1824744 | 2 | 2 | 0 | non-IDC | median | 3 | 2 | IIIA | 2 | 3 | 0 | 0.276135 | 1 | 18 |
| 71 | 0 | 1820976 | 3 | 3 | 1 | IDC | median | 2 | 2 | IIIA | 2 | 3 | 2 | 0.322594 | 1 | 43 |
| 72 | 0 | 1803370 | 1 | 2 | 0 | non-IDC | median | 2 | 2 | IIIA | 2 | 3 | 0 | 0.256946 | 1 | 29 |
| 73 | 0 | 1802079 | 3 | 2 | 1 | IDC | low | 3 | 2 | IIIA | 3 | 3 | 0 | 0.210827 | 1 | 38 |
| 74 | 0 | 1798777 | 3 | 3 | 1 | IDC | median | 2 | 3 | IIIC | 1 | 3 | 0 | 0.693313 | 1 | 44 |
| 75 | 0 | 1797018 | 2 | 2 | 0 | IDC | median | 4 | 3 | IIIC | 1 | 3 | 2 | 0.337459 | 1 | 7 |
| 76 | 0 | 1795460 | 3 | 1 | 1 | IDC | median | 2 | 2 | IIIA | 2 | 2 | 1 | 0.438627 | 1 | 47 |
| 77 | 0 | 1790979 | 3 | 3 | 1 | IDC | median | 1 | 2 | IIIA | 3 | 2 | 1 | 0.415144 | 1 | 16 |
| 78 | 0 | 1784009 | 1 | 2 | 0 | IDC | median | 2 | 1 | IIB | 2 | 3 | 1 | 0.063707 | 1 | 45 |
| 79 | 1 | 1755009 | 2 | 3 | 1 | IDC | median | 1 | 1 | IIA | 1 | 3 | 2 | 0.116137 | 1 | 48 |
| 80 | 0 | 1754103 | 2 | 2 | 1 | IDC | median | 3 | 3 | IIIC | 2 | 3 | 2 | 0.112652 | 1 | 19 |
| 81 | 0 | 1755516 | 1 | 3 | 0 | non-IDC | median | 3 | 1 | IIIA | 2 | 3 | 1 | 0.12947 | 1 | 20 |
| 82 | 0 | 1740744 | 2 | 3 | 0 | IDC | median | 1 | 1 | IIA | 1 | 3 | 1 | 0.296993 | 1 | 46 |
| 83 | 0 | 1746992 | 2 | 2 | 1 | IDC | median | 4 | 3 | IIIC | 3 | 3 | 1 | 0.450271 | 1 | 39 |
| 84 | 0 | 1737087 | 3 | 2 | 1 | IDC | median | 2 | 3 | IIIC | 1 | 3 | 2 | 0.495467 | 1 | 8 |
| 85 | 0 | 1731282 | 1 | 2 | 0 | IDC | median | 2 | 3 | IIIC | 2 | 1 | 1 | 0.069494 | 1 | 27 |
| 86 | 0 | 1715657 | 1 | 2 | 0 | IDC | median | 1 | 3 | IIIC | 2 | 2 | 1 | 0.11941 | 1 | 48 |
| 87 | 0 | 1718782 | 3 | 2 | 1 | IDC | median | 2 | 3 | IIIC | 2 | 3 | 1 | 0.555244 | 1 | 31 |
| 88 | 0 | 1709296 | 2 | 2 | 1 | IDC | median | 2 | 2 | IIIA | 1 | 3 | 2 | 0.321153 | 1 | 24 |
| 89 | 0 | 1681798 | 3 | 2 | 1 | IDC | high | 2 | 1 | IIB | 1 | 3 | 1 | 0.361127 | 1 | 1 |
| 90 | 0 | 1652017 | 1 | 2 | 0 | IDC | median | 2 | 1 | IIB | 3 | 3 | 1 | 0.079249 | 1 | 17 |
| 91 | 0 | 1595719 | 1 | 3 | 0 | IDC | median | 3 | 2 | IIIA | 3 | 3 | 1 | 0.323312 | 1 | 14 |
| 92 | 0 | 1526451 | 1 | 2 | 0 | IDC | median | 2 | 3 | IIIC | 2 | 3 | 1 | 0.270073 | 1 | 6 |
| 93 | 0 | 1511999 | 3 | 2 | 1 | IDC | median | 3 | 3 | IIIC | 3 | 3 | 2 | 0.290195 | 1 | 35 |
| 94 | 0 | 1509057 | 1 | 3 | 0 | IDC | median | 3 | 2 | IIIA | 3 | 3 | 1 | 0.323312 | 1 | 2 |
| 95 | 0 | 1477031 | 3 | 3 | 1 | non-IDC | low | 4 | 3 | IIIC | 1 | 3 | 2 | 0.24209 | 1 | 26 |
| 96 | 0 | 1440214 | 2 | 2 | 1 | IDC | median | 4 | 1 | IIIB | 3 | 3 | 2 | 0.204387 | 1 | 11 |

**PSM:** propensity score matching; **ID:** identity; **ER:** estrogen receptor; **IDC:** invasive ductal carcinoma.

**Supplementary Table 2.** Demographic and clinicopathological characteristics of patients with reduced sensitivity to chemotherapy

| **before PSM** | | | | | **after PSM** | | | |
| --- | --- | --- | --- | --- | --- | --- | --- | --- |
|  | **Neoadjuvant endocrine therapy**  **N=35** | **Neoadjuvant chemotherapy**  **N=119** | ***p*** | **Neoadjuvant endocrine therapy**  **N=34** | | **Neoadjuvant chemotherapy**  **N=34** | ***p*** |  |
| **Age at first diagnose** |  |  | **0.023** |  | |  | **0.779** |  |
| ≤45 | 8 (22.9%, 38y-45y) | 58 (48.7%, 29y-45y) |  | 8 (23.5%, 38y-45y) | | 6 (17.6%, 29y-45y) |  |  |
| 46-59 | 16 (45.7%, 49y-59y) | 38 (31.9%, 46y-59y) |  | 15 (44.1%, 49y-59y) | | 15 (44.1%, 47y-59y) |  |  |
| ≥60 | 11 (31.4%, 60y-72y) | 23 (19.3%, 60y-71y) |  | 11 (32.4%, 60y-72y) | | 13 (38.2%, 60y-71y) |  |  |
| **Menarche age** |  |  | **0.264** |  | |  | **1.000** |  |
| ≤12 | 2 (5.7%, 12y) | 20 (16.8%, 11y-12y) |  | 2 (5.9%, 12y) | | 3 (8.8%, 11y-12y) |  |  |
| 13-15 | 25 (71.4%, 13y-15y) | 75 (63.0%, 13y-15y) |  | 24 (70.6%, 13y-15y) | | 24 (70.6%, 13y-15y) |  |  |
| ≥16 | 8 (22.9%, 16y-19y) | 24 (20.2%, 16y-18y) |  | 8 (23.5%, 16y-19y) | | 7 (20.6%, 16y-18y) |  |  |
| **Menopause** |  |  | **0.053** |  | |  | **0.609** |  |
| yes | 21 (60.0%) | 48 (40.3%) |  | 21 (61.8%) | | 24 (70.6%) |  |  |
| no | 14 (40.0%) | 71 (59.7%) |  | 13 (38.2%) | | 10 (29.4%) |  |  |
| **Histology** |  |  | **0.662** |  | |  | **1.000** |  |
| IDC | 30 (85.7%) | 105 (88.2%) |  | 30 (88.2%) | | 30 (88.2%) |  |  |
| non-IDC | 5 (14.3%) | 13 (10.9%) |  | 4 (11.8%) | | 4 (11.8%) |  |  |
| NA | 0 (0.0%) | 1 (0.8%) |  | 0 (0.0%) | | 0 (0.0%) |  |  |
| **Grade** |  |  | **0.199** |  | |  | **1.000** |  |
| I | 1 (2.9%) | 2 (1.7%) |  | 1 (2.9%) | | 0 (0.0%) |  |  |
| II | 31 (88.6%) | 96 (80.7%) |  | 31 (91.2%) | | 31 (91.2%) |  |  |
| III | 2 (5.7%) | 20 (16.8%) |  | 2 (5.9%) | | 3 (8.8%) |  |  |
| others or NA | 1 (2.9%) | 1 (0.8%) |  | 0 (0.0%) | | 0 (0.0%) |  |  |
| **T stage (tumor size, mm)** |  |  | **0.229** |  | |  | **1.000** |  |
| 1 (T≤20) | 4 (11.4%) | 11 (9.2%) |  | 4 (11.8%) | | 3 (8.8%) |  |  |
| 2 (20<T≤50) | 16 (45.7%) | 60 (50.4%) |  | 15 (44.1%) | | 16 (47.1%) |  |  |
| 3 (T>50) | 8 (22.9%) | 38 (31.9%) |  | 8 (23.5%) | | 9 (26.5%) |  |  |
| 4 (inflammatory BC) | 7 (20.0%) | 10 (8.4%) |  | 7 (20.6%) | | 6 (17.6%) |  |  |
| **N stage** |  |  | **0.038** |  | |  | **0.885** |  |
| 0 | 1 (2.9%) | 4 (3.4%) |  | 1 (2.9%) | | 0 (0.0%) |  |  |
| 1 | 4 (11.4%) | 41 (34.5%) |  | 4 (11.8%) | | 4 (11.8%) |  |  |
| 2 | 18 (51.4%) | 40 (33.6%) |  | 17 (50.0%) | | 15 (44.1%) |  |  |
| 3 | 12 (34.3%) | 34 (28.6%) |  | 12 (35.3%) | | 15 (44.1%) |  |  |
| **Stage** |  |  | **0.038** |  | |  | **0.880** |  |
| I A | 0 (0.0%) | 0 (0.0%) |  | 0 (0.0%) | | 0 (0.0%) |  |  |
| II A | 2 (5.7%) | 8 (6.7%) |  | 2 (5.9%) | | 1 (2.9%) |  |  |
| II B | 1 (2.9%) | 23 (19.3%) |  | 1 (2.9%) | | 1 (2.9%) |  |  |
| III A | 15 (42.9%) | 49 (41.2%) |  | 14 (41.2%) | | 14 (41.2%) |  |  |
| III B | 5 (14.3%) | 5 (4.2%) |  | 5 (14.7%) | | 3 (8.8%) |  |  |
| III C | 12 (34.3%) | 34 (28.6%) |  | 12 (35.3%) | | 15 (44.1%) |  |  |
| **Ki67 level at baseline (%)** |  |  | **0.935** |  | |  | **0.660** |  |
| <15 | 7 (20.0%) | 20 (16.8%) |  | 7 (20.6%) | | 5 (14.7%) |  |  |
| 15-30 | 15 (42.9%) | 54 (45.4%) |  | 15 (44.1%) | | 14 (41.2%) |  |  |
| >30 | 13 (37.1%) | 45 (37.8%) |  | 12 (35.3%) | | 15 (44.1%) |  |  |
| **ER level at baseline (%)** |  |  | **0.108** |  | |  | **1.000** |  |
| ≤50 | 0 (0.0%) | 9 (7.6%) |  | 0 (0.0%) | | 0 (0.0%) |  |  |
| 51-80 | 9 (25.7%) | 41 (34.5%) |  | 8 (23.5%) | | 8 (23.5%) |  |  |
| ≥81 | 26 (74.3%) | 69 (58.0%) |  | 26 (76.5%) | | 26 (76.5%) |  |  |
| **HER2 status at baseline** |  |  | **0.304** |  | |  | **0.374** |  |
| 0 | 5 (14.3%) | 27 (22.7%) |  | 5 (14.7%) | | 6 (17.6%) |  |  |
| 1+ | 18 (51.4%) | 44 (37.0%) |  | 18 (52.9%) | | 12 (35.3%) |  |  |
| 2+FISH- | 12 (34.3%) | 48 (40.3%) |  | 11 (32.4%) | | 16 (47.1%) |  |  |

**PSM:** propensity score matching; **IDC:** invasive ductal carcinoma; **NA:** not available; **BC:** breast cancer; **ER:** estrogen receptor; **HER2:** human epidermal growth factor receptor 2.

**Supplementary Table 3.** PSM2 list

|  | **treatment** | **ID** | **age** | **menarche age** | **menopause** | **type** | **grade** | **T** | **N** | **stage** | **Ki67** | **ER** | **HER2** | **distance** | **weights** | **subclass** |
| --- | --- | --- | --- | --- | --- | --- | --- | --- | --- | --- | --- | --- | --- | --- | --- | --- |
| 1 | 1 | 1854110 | 2 | 2 | 1 | IDC | median | 2 | 2 | IIIA | 1 | 2 | 0 | 0.276807 | 1 | 1 |
| 2 | 1 | 1056883 | 3 | 2 | 1 | non-IDC | high | 4 | 2 | IIIB | 1 | 3 | 1 | 0.881013 | 1 | 2 |
| 3 | 1 | 1001051366 | 3 | 2 | 1 | IDC | median | 4 | 1 | IIIB | 2 | 3 | 1 | 0.691486 | 1 | 3 |
| 4 | 1 | 1926609 | 2 | 2 | 1 | IDC | median | 4 | 2 | IIIB | 2 | 3 | 1 | 0.960365 | 1 | 4 |
| 5 | 1 | 1857151 | 1 | 2 | 0 | IDC | median | 2 | 3 | IIIC | 2 | 3 | 1 | 0.308976 | 1 | 5 |
| 6 | 1 | 1879154 | 2 | 2 | 1 | IDC | median | 2 | 0 | IIA | 2 | 3 | 1 | 0.550972 | 1 | 6 |
| 7 | 1 | 1906651 | 3 | 2 | 1 | IDC | median | 3 | 2 | IIIA | 3 | 3 | 1 | 0.668248 | 1 | 7 |
| 8 | 1 | 1921661 | 2 | 2 | 1 | non-IDC | median | 3 | 2 | IIIA | 3 | 3 | 1 | 0.529611 | 1 | 8 |
| 9 | 1 | 1926429 | 3 | 2 | 1 | IDC | median | 2 | 2 | IIIA | 2 | 3 | 2 | 0.407686 | 1 | 9 |
| 10 | 1 | 1974922 | 2 | 3 | 1 | IDC | median | 1 | 3 | IIIC | 2 | 3 | 0 | 0.57713 | 1 | 10 |
| 11 | 1 | 1957391 | 1 | 2 | 0 | IDC | median | 2 | 2 | IIIA | 3 | 2 | 1 | 0.244503 | 1 | 11 |
| 12 | 1 | 1964028 | 2 | 3 | 1 | IDC | median | 2 | 2 | IIIA | 2 | 2 | 1 | 0.418501 | 1 | 12 |
| 13 | 1 | 1969561 | 1 | 2 | 0 | IDC | median | 2 | 3 | IIIC | 1 | 2 | 1 | 0.151829 | 1 | 13 |
| 14 | 1 | 1789815 | 3 | 3 | 1 | IDC | low | 4 | 3 | IIIC | 2 | 3 | 1 | 0.196279 | 1 | 14 |
| 15 | 1 | 1776627 | 1 | 3 | 0 | non-IDC | median | 3 | 3 | IIIC | 3 | 3 | 2 | 0.09607 | 1 | 15 |
| 16 | 1 | 1823375 | 1 | 1 | 0 | IDC | median | 3 | 2 | IIIA | 3 | 3 | 2 | 0.170613 | 1 | 16 |
| 17 | 1 | 1866867 | 2 | 2 | 0 | IDC | median | 4 | 2 | IIIB | 1 | 2 | 1 | 0.906547 | 1 | 17 |
| 18 | 1 | 1840635 | 2 | 3 | 1 | IDC | median | 2 | 2 | IIIA | 1 | 3 | 2 | 0.346386 | 1 | 18 |
| 19 | 1 | 1939921 | 2 | 3 | 0 | IDC | median | 2 | 2 | IIIA | 1 | 3 | 2 | 0.346386 | 1 | 19 |
| 20 | 1 | 1980918 | 3 | 2 | 1 | IDC | median | 2 | 3 | IIIC | 2 | 3 | 1 | 0.604344 | 1 | 20 |
| 21 | 1 | 1985960 | 3 | 2 | 1 | non-IDC | median | 4 | 3 | IIIC | 2 | 2 | 2 | 0.128794 | 1 | 21 |
| 22 | 1 | 2023393 | 1 | 2 | 0 | IDC | median | 3 | 2 | IIIA | 3 | 3 | 0 | 0.254364 | 1 | 22 |
| 23 | 1 | 2015365 | 1 | 1 | 0 | IDC | median | 1 | 2 | IIIA | 3 | 3 | 0 | 0.442183 | 1 | 23 |
| 24 | 1 | 2056243 | 2 | 2 | 1 | IDC | median | 2 | 2 | IIIA | 3 | 3 | 2 | 0.425877 | 1 | 24 |
| 25 | 1 | 2043250 | 3 | 2 | 1 | IDC | median | 3 | 2 | IIIA | 2 | 3 | 2 | 0.353235 | 1 | 25 |
| 26 | 0 | 2054954 | 2 | 2 | 0 | IDC | median | 2 | 2 | IIIA | 1 | 3 | 2 | 0.346386 | 1 | 25 |
| 27 | 0 | 1859014 | 2 | 2 | 1 | IDC | median | 2 | 3 | IIIC | 3 | 3 | 1 | 0.622094 | 1 | 17 |
| 28 | 0 | 2009922 | 2 | 2 | 0 | IDC | low | 1 | 3 | IIIC | 3 | 3 | 2 | 0.150647 | 1 | 13 |
| 29 | 0 | 1997414 | 3 | 1 | 1 | IDC | median | 4 | 3 | IIIC | 3 | 3 | 2 | 0.395581 | 1 | 12 |
| 30 | 0 | 1995232 | 1 | 2 | 0 | IDC | median | 2 | 2 | IIIA | 3 | 3 | 2 | 0.205873 | 1 | 31 |
| 31 | 1 | 1970683 | 2 | 2 | 1 | IDC | median | 3 | 3 | IIIC | 2 | 3 | 1 | 0.503767 | 1 | 26 |
| 32 | 0 | 1968526 | 3 | 2 | 1 | IDC | median | 1 | 1 | IIA | 1 | 3 | 1 | 0.341702 | 1 | 18 |
| 33 | 1 | 1866995 | 3 | 3 | 1 | IDC | median | 2 | 1 | IIB | 3 | 3 | 0 | 0.089113 | 1 | 27 |
| 34 | 1 | 1948704 | 3 | 2 | 1 | IDC | low | 1 | 3 | IIIC | 3 | 3 | 1 | 0.377714 | 1 | 28 |
| 35 | 0 | 1932072 | 2 | 2 | 1 | IDC | median | 3 | 3 | IIIC | 3 | 3 | 2 | 0.313047 | 1 | 32 |
| 36 | 0 | 1930626 | 3 | 2 | 1 | non-IDC | median | 3 | 3 | IIIC | 2 | 3 | 1 | 0.447154 | 1 | 8 |
| 37 | 1 | 1930502 | 3 | 2 | 1 | IDC | median | 2 | 3 | IIIC | 3 | 3 | 1 | 0.662771 | 1 | 29 |
| 38 | 0 | 1920042 | 3 | 3 | 1 | IDC | median | 2 | 2 | IIIA | 2 | 3 | 2 | 0.407686 | 1 | 24 |
| 39 | 0 | 1854810 | 3 | 2 | 1 | IDC | median | 4 | 3 | IIIC | 2 | 3 | 2 | 0.337159 | 1 | 19 |
| 40 | 1 | 1921024 | 2 | 2 | 0 | IDC | median | 2 | 3 | IIIC | 2 | 2 | 1 | 0.35782 | 1 | 30 |
| 41 | 1 | 1918277 | 2 | 2 | 0 | IDC | median | 4 | 1 | IIIB | 2 | 2 | 2 | 0.221937 | 1 | 31 |
| 42 | 1 | 1910117 | 2 | 2 | 0 | IDC | median | 3 | 2 | IIIA | 2 | 3 | 2 | 0.313874 | 1 | 32 |
| 43 | 0 | 1890346 | 2 | 2 | 1 | IDC | median | 2 | 2 | IIIA | 2 | 2 | 1 | 0.418501 | 1 | 26 |
| 44 | 0 | 1890190 | 2 | 2 | 1 | IDC | median | 2 | 3 | IIIC | 2 | 3 | 1 | 0.561285 | 1 | 3 |
| 45 | 0 | 1836158 | 3 | 3 | 1 | IDC | low | 2 | 2 | IIIA | 3 | 3 | 2 | 0.129177 | 1 | 21 |
| 46 | 0 | 1837107 | 1 | 2 | 0 | IDC | median | 3 | 3 | IIIC | 2 | 3 | 0 | 0.170307 | 1 | 16 |
| 47 | 0 | 1829291 | 2 | 3 | 0 | IDC | median | 2 | 2 | IIIA | 1 | 3 | 0 | 0.46776 | 1 | 7 |
| 48 | 0 | 1850082 | 3 | 2 | 1 | IDC | median | 1 | 2 | IIIA | 3 | 3 | 1 | 0.823964 | 1 | 4 |
| 49 | 1 | 1898553 | 1 | 2 | 0 | IDC | median | 2 | 3 | IIIC | 3 | 3 | 1 | 0.365205 | 1 | 33 |
| 50 | 0 | 1949680 | 3 | 2 | 1 | IDC | median | 2 | 1 | IIB | 3 | 3 | 0 | 0.089113 | 1 | 27 |
| 51 | 0 | 1813388 | 2 | 2 | 1 | non-IDC | median | 3 | 3 | IIIC | 2 | 2 | 1 | 0.227828 | 1 | 11 |
| 52 | 0 | 1804957 | 3 | 2 | 1 | non-IDC | median | 2 | 2 | IIIA | 3 | 3 | 2 | 0.371464 | 1 | 9 |
| 53 | 0 | 1798777 | 3 | 3 | 1 | IDC | median | 2 | 3 | IIIC | 1 | 3 | 0 | 0.448229 | 1 | 6 |
| 54 | 0 | 1795460 | 3 | 1 | 1 | IDC | median | 2 | 2 | IIIA | 2 | 2 | 1 | 0.462146 | 1 | 29 |
| 55 | 0 | 1786098 | 2 | 2 | 0 | IDC | median | 2 | 3 | IIIC | 3 | 2 | 1 | 0.417567 | 1 | 23 |
| 56 | 0 | 1749183 | 3 | 3 | 1 | IDC | median | 3 | 2 | IIIA | 2 | 2 | 2 | 0.192155 | 1 | 14 |
| 57 | 1 | 1755009 | 2 | 3 | 1 | IDC | median | 1 | 1 | IIA | 1 | 3 | 2 | 0.131702 | 1 | 34 |
| 58 | 0 | 1754103 | 2 | 2 | 1 | IDC | median | 3 | 3 | IIIC | 2 | 3 | 2 | 0.26154 | 1 | 22 |
| 59 | 0 | 1755422 | 1 | 1 | 0 | IDC | median | 4 | 1 | IIIB | 2 | 3 | 0 | 0.275146 | 1 | 1 |
| 60 | 0 | 1750612 | 2 | 2 | 1 | IDC | median | 3 | 3 | IIIC | 2 | 2 | 2 | 0.133634 | 1 | 34 |
| 61 | 0 | 1746992 | 2 | 2 | 1 | IDC | median | 4 | 3 | IIIC | 3 | 3 | 1 | 0.611095 | 1 | 2 |
| 62 | 0 | 1709296 | 2 | 2 | 1 | IDC | median | 2 | 2 | IIIA | 1 | 3 | 2 | 0.346386 | 1 | 30 |
| 63 | 0 | 1699121 | 3 | 3 | 1 | IDC | low | 2 | 2 | IIIA | 3 | 2 | 0 | 0.096769 | 1 | 15 |
| 64 | 0 | 1684821 | 1 | 2 | 0 | non-IDC | median | 4 | 2 | IIIB | 2 | 2 | 2 | 0.461966 | 1 | 20 |
| 65 | 0 | 1526451 | 1 | 2 | 0 | IDC | median | 2 | 3 | IIIC | 2 | 3 | 1 | 0.308976 | 1 | 5 |
| 66 | 0 | 1509057 | 1 | 3 | 0 | IDC | median | 3 | 2 | IIIA | 3 | 3 | 1 | 0.370927 | 1 | 28 |
| 67 | 0 | 1440214 | 2 | 2 | 1 | IDC | median | 4 | 1 | IIIB | 3 | 3 | 2 | 0.457322 | 1 | 10 |
| 68 | 0 | 1429616 | 2 | 2 | 1 | IDC | median | 3 | 2 | IIIA | 3 | 3 | 2 | 0.370517 | 1 | 33 |

**PSM:** propensity score matching; **ID:** identity; **ER:** estrogen receptor; **IDC:** invasive ductal carcinoma.
